# Contributions of Sex Chromosomes and Gonadal Hormones to the Male Bias in a Maternal Antibody-Induced Model of Autism Spectrum Disorder

**DOI:** 10.3389/fneur.2021.721108

**Published:** 2021-10-13

**Authors:** Adriana Gata-Garcia, Amit Porat, Lior Brimberg, Bruce T. Volpe, Patricio T. Huerta, Betty Diamond

**Affiliations:** ^1^Center for Autoimmune, Musculoskeletal and Hematopoietic Diseases, Institute of Molecular Medicine, Feinstein Institutes for Medical Research, Northwell Health, Manhasset, NY, United States; ^2^Donald and Barbara Zucker School of Medicine at Hofstra/Northwell, Hempstead, NY, United States; ^3^Elmezzi Graduate School of Molecular Medicine, Feinstein Institutes for Medical Research, Northwell Health, Manhasset, NY, United States; ^4^Laboratory of Immune and Neural Networks, Institute of Molecular Medicine, Feinstein Institutes for Medical Research, Northwell Health, Manhasset, NY, United States

**Keywords:** autism spectrum disorder, male bias, sex chromosome, gonadal hormones, four core genotypes, SRY gene, maternal antibody

## Abstract

Autism Spectrum Disorder (ASD) is a group of neurodevelopmental conditions that is four times more commonly diagnosed in males than females. While susceptibility genes located in the sex chromosomes have been identified in ASD, it is unclear whether they are sufficient to explain the male bias or whether gonadal hormones also play a key role. We evaluated the sex chromosomal and hormonal influences on the male bias in a murine model of ASD, in which mice are exposed *in utero* to a maternal antibody reactive to contactin-associated protein-like 2 (Caspr2), which was originally cloned from a mother of a child with ASD (termed C6 mice henceforth). In this model, only male mice are affected. We used the four-core-genotypes (FCG) model in which the *Sry* gene is deleted from the Y chromosome (Y^−^) and inserted into autosome 3 (*TgSry*). Thus, by combining the C6 and FCG models, we were able to differentiate the contributions of sex chromosomes and gonadal hormones to the development of fetal brain and adult behavioral phenotypes. We show that the presence of the Y chromosome, or lack of two X chromosomes, irrespective of gonadal sex, increased the susceptibility to C6-induced phenotypes including the abnormal growth of the developing fetal cerebral cortex, as well as a behavioral pattern of decreased open-field exploration in adult mice. Our results indicate that sex chromosomes are the main determinant of the male bias in the maternal C6-induced model of ASD. The less dominant hormonal effect may be due to modulation by sex chromosome genes of factors involved in gonadal hormone pathways in the brain.

## Introduction

Autism Spectrum Disorder (ASD) is a group of neurodevelopmental conditions that manifest early in childhood. ASD is characterized by varying degrees of impairment in social interaction and communication, and by restricted interests and repetitive behaviors (DSM-V). In the U.S., about 1 in 59 children was diagnosed with ASD in 2014 (1). Both genetic and environmental factors contribute to the etiology of ASD [reviewed in (2)]. A recent study estimated the heritability of ASD to be 80% (3). However, environmental factors, especially those present during critical periods of prenatal and perinatal brain development, play an essential role in modulating the risk to develop ASD and may account, in part, for the phenotypic variability observed (2).

ASD is four times more frequently diagnosed in males than in females (1). A strong male preponderance is not unique to ASD; other neuropsychiatric conditions, especially those diagnosed earlier in life, also show a male sex bias [reviewed in (4)]. Conversely, neuropsychiatric conditions with a female predominance, including anorexia nervosa and internalizing disorders such as depression and anxiety, present during puberty or later in life [reviewed in (4–6)]. Identifying factors that contribute to neuropsychiatric syndrome susceptibility in either males or females will increase our understanding of brain development and the pathogenesis of these conditions while also providing a foundation for the discovery of new treatments.

Maternal brain-reactive antibodies and gonadal hormones include some of the environmental factors *in utero* that may contribute to ASD risk and its sex bias. This study evaluated the role of gonadal hormones and sex chromosomes in the male bias we observed in a maternal antibody-induced mouse model of ASD (7). In this model, mice were exposed *in utero* to C6, a monoclonal antibody reactive to contactin-associated protein-like 2 (Caspr2) cloned from a mother of a child with ASD, or to an isotype matched control antibody, B1. Caspr2 is a cell-adhesion molecule expressed by neurons (8, 9). Rare and common variants of *CNTNAP2*, the gene encoding Caspr2, have been linked to an increased risk of ASD or ASD-related phenotypes including language delay and developmental language disorders (10–20). *In utero* exposure to C6, but not to B1, leads to thinning of the cortical plate (CP), impaired social interactions, and increased repetitive behaviors in male offspring only in C57BL/6 mice (7). Here, we used the four-core-genotypes (FCG) mouse model to isolate gonadal hormone from sex chromosome contributions to the male bias observed (21). The FCG model allows for the dissociation of gonadal development from sex chromosome complement through the deletion of the *Sry* gene from the Y chromosome (referred to as Y^−^) and the insertion of the *Sry* transgene into autosome 3 (*TgSry*), leading to the development of mice with four genotypes: *XY*^−^ *TgSry, XX TgSry, XY*^−^, and *XX* (21, 22), with the first two having and the second two lacking male hormone synthesis *in utero*. Our results demonstrate that the male bias of thinner CP seen during fetal development is driven by sex chromosome differences. Furthermore, we found that, in this strain of mice, *in utero* exposure to C6 antibody causes increased sustained anxiety-like behavior which may also be sex chromosome dependent.

## Materials and Methods

### Ethical Statement

All animal experimentation was performed in accordance with the National Institutes of Health (NIH) Guidelines, under protocols approved by the Institutional Animal Care and Use Committee (IACUC) of the Feinstein Institutes for Medical Research.

### *In vitro* Antibody Production

As described previously (7), human embryonic kidney fibroblast 293T cells (HEK-293T, ATCC CRL 11268^TM^) were split into culture dishes (100 × 20 mm) in high glucose DMEM (HyClone, GE Healthcare), supplemented with heat inactivated fetal bovine serum (FBS, 10%), glutamine (1%) and penicillin-streptomycin (1%, HyClone, GE Healthcare). After 16–24 h, at 70–80% confluence, the medium was replaced with SFM4Transfx-293 (Hyclone, GE Healthcare) supplemented with 10% FBS, 1% glutamine and 1% penicillin-streptomycin (HyClone, GE Healthcare). Cells were co-transfected 8 h later, using Lipofectamine LTX Reagent (Invitrogen), with IgH and IgL encoding plasmids (5 μg). Supernatants were collected after 7 days, and the antibodies were purified on protein G-sepharose beads (GE Healthcare, Life Technologies). Glycine buffer (0.1 M, pH 3.5) and Tris-HCl (1 M, pH 8) were used for antibody elution and pH neutralization, respectively. Purified antibody was dialyzed in PBS and its concentration was determined by Nanodrop. Antibody integrity was assessed on NuPAGE 4–12% BisTris gels (Invitrogen) stained with Coomassie blue.

### Timed Pregnancies and Antibody Administration

**Figure 1:**
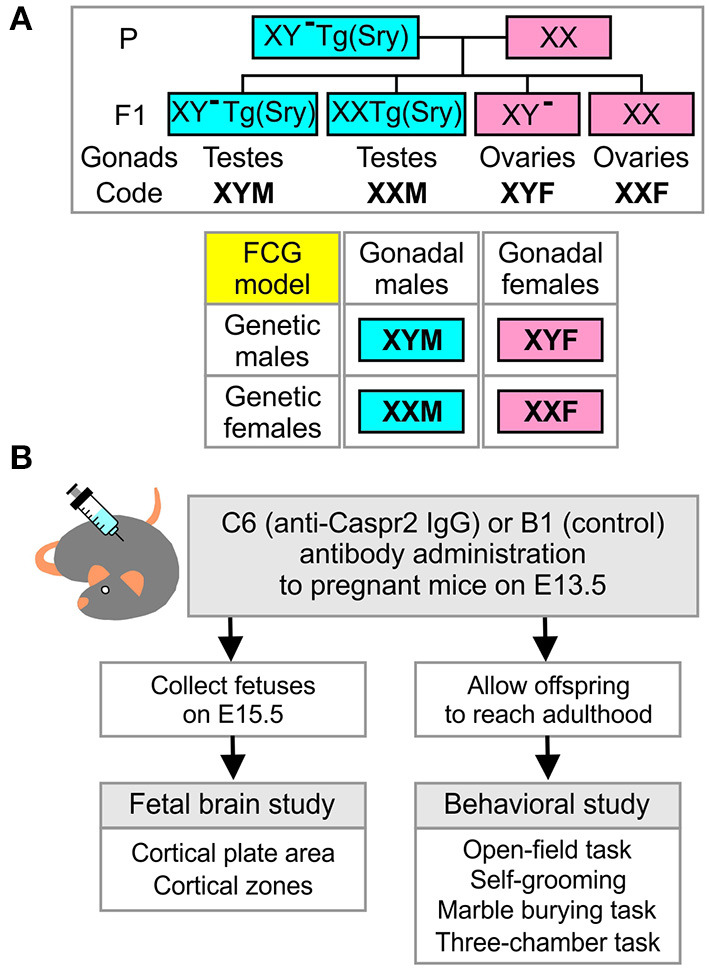
Four-core-genotypes model, combined with *in utero* exposure to C6, to study maternal antibody-induced model of ASD. **(A)** Description of the four-core-genotypes (FCG) model in which a C57Bl/6J female mouse (XX) is mated with a XY^−^(*Sry*) male mouse (XYM) to generate offspring in which gonadal and chromosomal contributions can be separately studied. **(B)** Description of the experimental plan for the fetal brain and behavioral studies.

C57BL/6 female mice and *XY*^−^ *TgSry* male mice (6–10 weeks old) obtained from the Jackson Laboratory were used for timed pregnancies. In detail, two wild type females and one *XY*^−^ *TgSry* male mouse were housed together for 15 h starting at the beginning of the dark cycle. The male was removed after 15 h. This mating scheme produced offspring with the genotypes *XY*^−^ *TgSry* (termed XYM henceforth), *XX TgSry* (termed XXM), *XY*^−^ (termed XYF), and *XX* (termed XXF) (Figure 1A). Embryonic age was determined by the time of male mouse removal which was defined as embryonic day E0.5. Pregnant females were randomly assigned to receive either C6 (anti-Caspr2 IgG, 200 μg) or B1 (control IgG, 200 μg) by retro-orbital sinus injection under light isoflurane anesthesia on E13.5. Subsequently, E15.5 embryos were harvested, genotyped, and processed for brain histology (Figure 1B). In the fetal analysis, 5–7 litters per antibody were included. No more than two fetuses per genotype were selected from each litter for analysis. When two mice were derived from the same litter, the average score was used. A subgroup of pregnancies was assigned for offspring behavior assessment and allowed to reach full term (Figure 1B). Adult mice from each of the four core genotypes (FCG) were obtained from eight litters for C6 and six litters for B1.

### Fetal Processing for Brain Histological Analysis

Fetuses were fixed with a 4% paraformaldehyde, 4% sucrose solution for 4 h, at 4°C. They were then cryoprotected at 4°C with a gradual increase in sucrose (45 min in 10% sucrose, 1 h in 20% sucrose, and overnight in 30% sucrose). Fetuses were then submerged in a 1:1 solution of 30% sucrose and OCT compound (Fisher Scientific, Pittsburg, PA, USA) for 1 h at room temperature (RT) and transferred to a cryomold with OCT and frozen on dry ice. Samples were kept at −80°C until cut. Whole embryos were mounted and cut with a Cryostat (Leica, Billerica, MA, USA) and all sections (14 μm) were collected and mounted on gelatin-coated slides. The sampling strategy ensured that sagittal sections 8 and 9, according to the Prenatal Mouse Brain Atlas [(23), p. 295–7], were included. Sections were stored at −80°C until stained.

### Fetal Brain Immunohistochemistry

Sections were thawed for 25 min and rinsed once with 1 × PBS at RT. For antigen retrieval, sections were heated in 10 mM Sodium Citrate Buffer for 10 min at 95°C and cooled to RT with two rinses of 1 × PBS. Blocking was done for 1 h, at RT, with a blocking buffer containing 1 × PBS/0.1% Triton X-100/3% normal goat serum/2.5% bovine serum albumin (BSA). Sections were then incubated with anti-nestin antibody (MAB353, Millipore, Wetzlar, Germany), at 1:400 dilution in 1 × PBS/0.1% Triton X-100/5% BSA, overnight at 4°C. After washing with 1 × PBS/0.1% Tween20, Alexa Fluor 488 goat anti-mouse IgG (A11001, Life Technologies), at 1:400 dilution in 1 × PBS with 0.2% BSA, was used to detect antibody binding (45 min incubation at RT). Secondary antibody was washed off with 1 × PBS/0.1% Tween20, and sections were stained with DAPI (Life Technologies) for 7 min at RT. Sections were washed with 1 × PBS, and coverslipped with Dako Fluorescence Mounting Medium (Dako North America Inc.). Images were obtained with Axio-Imager (Z-1, Zen3.1, Zeiss, Peabody, MA, USA).

### Analysis of Cortical Plate and Cortical Zones

**Figure 2:**
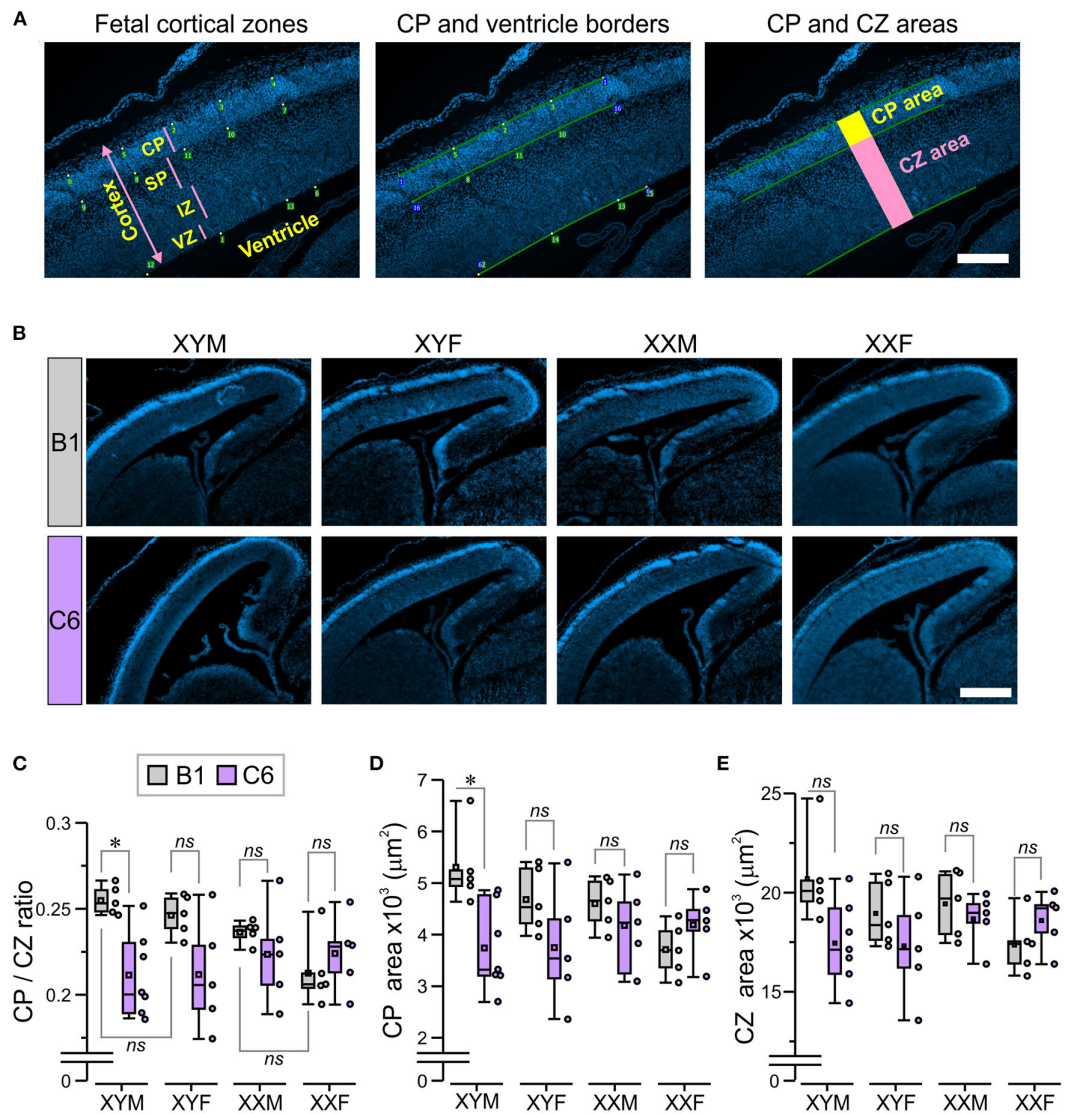
The presence of the Y chromosome predisposes fetuses to develop a smaller cortical plate due to C6 exposure *in utero*. We established mating pairs between wild type C57Bl/6J female and XYM male mice. This mating scheme produced offspring with four genotypes, whose gonadal development was independent of sex chromosome complement. Offspring from 5 to 7 litters for each antibody were analyzed, with no more than two offspring per genotype selected from each litter. **(A)** The micrographs describe the technique for measuring the cortical plate (CP) area and the CZ area, which included the subplate (SP), intermediate zone (IZ), and ventricular zone (VZ; as explained in detail in the Methods. Scale = 100 μm. **(B)** The panels show staining with DAPI, which was used to identify the CP in E15.5 fetuses. Scale = 400 μm. **(C–E)** Box-and-whisker plots represent median and Q1–Q3 quartiles (whiskers are 10–90 range). Dots represent individual measurements. **(C)** Quantification of CP/CZ ratio shows that C6-exposed XYM fetuses had a smaller ratio relative to their respective B1-exposed controls. **(D)** Measurement of the CP area. Compared to their B1 control fetuses, C6-XYM fetuses had a significantly smaller CP area. **(E)** Measurement of the CZ area. There were no significant differences in the cortical area between C6 and B1 exposed mice irrespective of genotype; 2-way ANOVA with Tukey test was used for statistical comparisons. See Table 1 and Supplementary Table 1 for details of statistical testing; *ns*, non-significant; *P < 0.05.

The cortical plate (CP) was identified as the highly DAPI-dense band of cells, most distal from the ventricle. This was confirmed on the alternate slides, stained with nestin, which was absent in this region (24). Microscope images (10 × ) of fetal sections stained with DAPI were digitally analyzed using a program that generated orthogonal lines so that a 2-D cortical region was isolated for measurement (Figure 2A). The program allowed the placement of points along the borders of the CP and the ventricle, which were defined using the zoom function (Pyramid, Axiophot2.1, Zen 3.1, Zeiss), and the software joined the points. The CP area was measured as the region contained within the superior and inferior borders of the appropriately joined points and the orthogonal lines (Figure 2A). Next, the region containing the subplate (SP), intermediate zone (IZ), and ventricular zone (VZ) was measured, and defined as the ‘cortical zones' (CZ) area (Figure 2A); the marginal zone was not included in the CZ area. The CP/CZ ratio was calculated by dividing the two areas, followed by repeated sampling (20–30 × ) throughout the length of the defined CP within the section. Additionally, the VZ was defined by the presence of spindle-shaped cells on the edge of the tissue and bright DAPI stain, the IZ was distinguished as the densely-packed DAPI-stained cells (dorsal to VZ), whereas the SP was defined as the sparsely-packed region located dorsal to IZ and ventral to the DAPI-dense CP (Figure 2A). We evaluated 1–3 alternate sections per fetal brain with the CP area, CZ area, CP/CZ ratio, as well as the SP, IZ, and VZ areas being averaged for the entire section. The mean values were used for statistical comparisons across groups. The investigator that performed the CP analysis was blinded to both genotype and antibody exposure. A separate investigator performed the statistical analysis.

### Behavioral Assessments

FCG mice exposed to C6 or B1 IgG *in utero* were housed under a reversed dark (9:00–21:00) and light (21:00–9:00) cycle, with *ad libitum* access to food and water. All manipulations were conducted during the dark phase, at least 1 h after turning lights off, and male and female mice were assessed on different days. Prior to behavioral assessments, mice were handled three times, for 15 min each, on separate days. At 6–11 weeks of age, mice underwent an observational screen, which we have described in detail previously (24), to assess muscle and spinal, spinocerebellar, sensory, neuropsychiatric, and autonomic functions (**Table 2**). Behavioral assessments were performed at 14–26 weeks of age and included the open-field task (14–15 weeks of age), followed by the marble burying task (17–20 weeks of age), and finally the three-chamber task (22–26 weeks of age). Experiments were conducted and analyzed according to randomly assigned cage numbers which did not indicate mouse genotype or antibody exposure. The investigator was blinded to both genotype and antibody exposure.

### Open-Field Task

This task was used to examine locomotor activity, habituation to a novel chamber, and anxiety-like behavior by placing the mice in the center of a square arena (40 × 40 cm^2^) with gray walls (35 cm high) and allowing them to freely explore the chamber during two sessions (10 min each) separated by 24 h. The sessions were recorded with a centrally placed video camera directly above the arena which fed the signal to the tracking software (EthoVision XT 14.0, Noldus, Attleboro, MA, USA) used for automated analysis of animal behaviors including distance traveled, velocity, time spent moving, time spent in the center of the arena (18.9 × 18.9 cm^2^), and self-grooming. We used customized settings, within EthoVision, to detect only grooming bouts >2 s, thus reducing the detection of extra-short bouts.

### Marble Burying Task

Repetitive behavior was examined with the marble burying task (25) in which mice were placed in the center of a square arena (40 × 40 cm^2^, 35-cm wall height) containing 25 black glass marbles (1.2 cm diameter) placed on top of corn cob bedding (5 cm depth) in a 5 × 5 grid pattern arrangement. The mice freely explored the environment for 20 min and at the end of the session, the number of buried marbles (>50% of marble surface area covered by bedding material) was recorded.

### Three-Chamber Task

This task was used to measure social approach, by placing the subject mouse in an apparatus (60 cm × 40 cm, 40-cm wall height) with clear plexiglass walls, which was subdivided into 3 chambers with sealable doors between chambers. A subject mouse was placed in the center chamber with the left and right chambers sealed off; the doors were opened, and the subject was allowed to explore the empty arena, with access to the 3 chambers, for 10 min. The subject mouse was then gently guided to the center chamber and access to the side chambers was sealed off. An age and sex matched (XYM or XXF) unfamiliar “mouse-stimulus” was placed in one of the side chambers, confined by a cylinder (diameter 9 cm, height 20 cm) with the bottom 5.5 cm perforated with holes (diameter, 0.5 cm). The cylinder was 3D-printed with clear methacrylate resin (FormLabs, Somerville, MA, USA). Each mouse-stimulus was acclimated to the cylinder for 10 min prior to the experiment. An identical but empty methacrylate cylinder was used as a novel object and placed in the second side chamber. The subject mouse was then allowed to explore the arena for 10 min, with access to the three chambers. The chamber used for the mouse-stimulus and the novel object were alternated between trials. The three-chambered apparatus and the methacrylate cylinders were cleaned prior to each trial with 70% ethanol followed by water and wiped dry. Video tracking software (Etovision XT 14.0) was used to obtain the total time spent interacting with the mouse-stimulus and with the novel object. A discrimination ratio was computed by the formula (T_MS_-T_O_)/(T_MS_ + T_O_), in which T_MS_ denotes the time near the mouse-stimulus and T_O_ denotes the time near the novel object.

### Statistical Analysis

Most datasets were analyzed with two-way analysis of variance (ANOVA), with “genotype” (XYM, XXM, XYF and XXF) and “antibody” (C6 and B1) as factors, which was followed by *post-hoc* Tukey correction for multiple comparisons. For the open-field task, time series data were analyzed with 2-way repeated measures ANOVA (RMANOVA) followed by *post-hoc* Bonferroni correction. For the two sessions of the open-field task, data were analyzed with 3-way ANOVA (with genotype, antibody and sessions as factors) followed by Tukey correction for multiple comparisons. For the three-chamber task, 3-way ANOVA was also used, with genotype, antibody and “stimulus” (mouse-stimulus and object) as factors, followed by Tukey correction for multiple comparisons. The software Origin [Origin Pro 2021b (64-bit) SR2, OriginLab, Northampton, MA] was used for statistical tests. *P* < 0.05 were considered statistically significant.

## Results

### Effects of *in utero* Exposure to C6 on Cortical Thickness Depend on Sex Chromosome Complement

We established mating pairs between wild type C57Bl/6J female and *XY*^−^ *TgSry* male mice (Figure 1A), which delivered offspring with the genotypes *XY*^−^ *TgSry* (termed XYM), *XX TgSry* (termed XXM), *XY*^−^ (termed XYF), and *XX* (termed XXF) (Figure 1A). We administered intravenous C6 or B1 antibody to pregnant mice on embryonic day E13.5 and harvested the fetuses on day E15.5 (7) (Figure 1B). We used the XYM and XXF mice as internal controls to confirm that, as in wild type C57BL/6J mice, *in utero* exposure to C6 led to ASD-like phenotypes in males but not females in this mouse strain. Offspring from 5 to 7 litters for each antibody were analyzed, with no more than two offspring per genotype selected from each litter. Not all genotypes were present in all litters.

We measured the CP/CZ ratio (Figure 2A) in hormonal and chromosomal males and females exposed to C6 or B1, as we had shown this ratio to be diminished in C57BL/6 males, but not female mice exposed to C6 *in utero* (7). Crucially, we observed no effect of C6 administration in XXF offspring (Figures 2B,C, *P* > 0.1, Table 1) and a decrease in the CP/CZ ratio in XYM offspring (Figure 2C), replicating our previous results in C57BL/6 mice (7). Indeed, we found that C6-exposed XYM fetuses had a significantly smaller CP/CZ ratio when compared to B1-XYM control fetuses (Figure 2C; *q* = 4.89, *P* = 0.029, see Table 1 and Supplementary Table 1 for details on statistical tests), which was likely due to a decrease in CP area specifically and not to smaller SP, IZ or VZ. Of note, the C6-treated fetuses displayed high variability in the CP/CZ ratio and, predictably, two-way ANOVA revealed a significant effect for the “antibody” factor [C6 vs. B1 groups, *F*_(1, 41)_ = 9.1, *P* = 0.0048] and the antibody × genotype interaction [*F*_(3, 41)_ = 3.29, *P* = 0.03], while the *post-hoc* Tukey correction confirmed the C6 vs. B1 effect (*q* = 4.41, *P* = 0.004). However, the comparisons of the interactions for all the other groups failed to reach statistical significance (Supplementary Table 1).

**Table 1:** Statistical analysis for the parameters presented in the figures.

**Figure**	**Groups**	**mean ± SEM**	**Interactions**	**Statistic**	***P*-value**
Figure 2C	**CP/CZ ratio**		**2Way-ANOVA followed by Tukey test**		
	B1-XYM	0.255 ± 0.003	B1-XYM vs. C6-XYM	*q =* 4.892	0.029
	C6-XYM	0.211 ± 0.009	B1-XYF vs. C6-XYF	*q =* 3.565	0.22
	B1-XYF	0.246 ± 0.005	B1-XXM vs. C6-XXM	*q =* 1.38	0.97
	C6-XYF	0.212 ± 0.015	B1-XXF vs. C6-XXF	*q =* 1.096	0.99
	B1-XXM	0.237 ± 0.003	B1-XYM vs. B1-XYF	*q =* 0.907	0.99
	C6-XXM	0.223 ± 0.013	B1-XXM vs. B1-XXF	*q =* 2.392	0.692
	B1-XXF	0.213 ± 0.009			
	C6-XXF	0.224 ± 0.009			
Figure 2D	**CP area (μm** ^**2**^ **)**		**2Way-ANOVA followed by Tukey test**		
	B1-XYM	5,307.87 ± 341.59	B1-XYM vs. C6-XYM	*q =* 4.987	0.024
	C6-XYM	3,735.62 ± 317.91	B1-XYF vs. C6-XYF	*q =* 2.74	0.53
	B1-XYF	4,690.79 ± 287.51	B1-XXM vs. C6-XXM	*q =* 1.24	0.98
	C6-XYF	3,757.42 ± 516.34	B1-XXF vs. C6-XXF	*q =* 1.384	0.97
	B1-XXM	4,611.52 ± 221.75			
	C6-XXM	4,189.88 ± 354.11			
	B1-XXF	3,718.58 ± 231.27			
	C6-XXF	4,190.05 ± 282.18			
Figure 2E	**CZ area (μm** ^**2**^ **)**		**2Way-ANOVA followed by Tukey test**		
	B1-XYM	20,736.68 ± 1,049.35	B1-XYM vs. C6-XYM	*q =* 4.146	0.097
	C6-XYM	17,444.57 ± 789.28	B1-XYF vs. C6-XYF	*q =* 1.899	0.875
	B1-XYF	18,955.79 ± 754.52	B1-XXM vs. C6-XXM	*q =* 0.909	0.99
	C6-XYF	17,326.93 ± 1,216.2	B1-XXF vs. C6-XXF	*q =* 1.4	0.972
	B1-XXM	19,433.69 ± 752.77			
	C6-XXM	18,653.41 ± 605.75			
	B1-XXF	17,394.5 ± 665.74			
	C6-XXF	18,595.52 ± 645.57			
Figure 3B	**Open-field task: Mean time-in-center (s)**		**2Way-RMANOVA followed by Bonferroni test**		
	B1-XYM-S1	0.937 ± 0.054	S1: B1-XYM vs. C6-XYM	*F_(15)_* = 0.412	0.686
	B1-XYM-S2	2.07 ± 0.094	S2: B1-XYM vs. C6-XYM	*F_(15)_* = 6.285	1.462 × 10^−5^
	C6-XYM-S1	0.902 ± 0.064	S1: B1-XYF vs. C6-XYF	*F_(15)_* = 1.61	0.13
	C6-XYM-S2	1.148 ± 0.113	S2: B1-XYF vs. C6-XYF	*F_(15)_* = 9.384	1.145 × 10^−7^
	B1-XYF-S1	1.054 ± 6.97	S1: B1-XXM vs. C6-XXM	*F_(15)_* = 1.451	0.164
	B1-XYF-S2	1.886 ± 0.094	S2: B1-XXM vs. C6-XXM	*F_(15)_* = 1.337	0.198
	C6-XYF-S1	0.924 ± 0.052			
	C6-XYF-S2	0.731 ± 0.079			
	B1-XXM-S1	1.003 ± 0.101			
	B1-XXM-S2	1.674 ± 0.209			
	C6-XXM-S1	1.212 ± 0.101			
	C6-XXM-S2	2.069 ± 0.21			
Figure 3C	**OF task: total time-in center per subject (s)**		**3Way-ANOVA followed by Tukey test**		
	B1-XYM-S1	55.252 ± 6.12	B1-XYM-S2 vs. C6-XYM-S2	*q* = 5.473	0.01
	B1-XYM-S2	122.13 ± 16.23	B1-XYF-S2 vs. C6-XYF-S2	*q* = 6.857	2.886 × 10^−4^
	C6-XYM-S1	53.222 ± 7.45	B1-XXM-S2 vs. C6-XXM-S2	*q* = 2.591	0.796
	C6-XYM-S2	67.736 ± 11.1	C6-XYM-S1 vs. C6-XYM-S2	*q* = 1.346	0.998
	B1-XYF-S1	62.188 ± 6.97	C6-XYF-S1 vs. C6-XYF-S2	*q* = 1.265	0.999
	B1-XYF-S2	111.26 ± 16.55			
	C6-XYF-S1	54.518 ± 2.07			
	C6-XYF-S2	43.113 ± 7.74			
	B1-XXM-S1	59.202 ± 4.88			
	B1-XXM-S2	98.728 ± 14.03			
	C6-XXM-S1	71.482 ± 6.92			
	C6-XXM-S2	122.103 ± 10.43			
Figure 3D	**OF task: time difference [S2–S1] (s)**		**2Way-ANOVA followed by Tukey test**		
	B1-XYM	66.878 ± 13.688	B1-XYM vs. C6-XYM	*q =* 4.265	0.004
	C6-XYM	14.514 ± 11.555	B1-XYF vs. C6-XYF	*q =* 4.596	0.024
	B1-XYF	49.075 ± 19.05	B1-XXM vs. C6-XXM	*q =* 0.929	0.98
	C6-XYF	−11.405 ± 7.832			
	B1-XXM	39.525 ± 13.415			
	C6-XXM	50.621 ± 9.999			
Figure 4A	**OF task: time moving (s)**		**3Way-ANOVA followed by Tukey test**		
	B1-XYM-S1	370.17 ± 31.7	B1-XYM-S2 vs. C6-XYM-S2	*q* = 0.8455	0.99
	B1-XYM-S2	271.42 ± 12.22	B1-XYF-S2 vs. C6-XYF-S2	*q* = 0.138	0.99
	C6-XYM-S1	381.39 ± 23.51	B1-XXM-S2 vs. C6-XXM-S2	*q* = 0.811	0.99
	C6-XYM-S2	261.71 ± 11.13			
	B1-XYF-S1	340.31 ± 40.05			
	B1-XYF-S2	260.00 ± 11.01			
	C6-XYF-S1	335.75 ± 25.64			
	C6-XYF-S2	200.14 ± 15.24			
	B1-XXM-S1	335.45 ± 27.99			
	B1-XXM-S2	279.01 ± 10.65			
	C6-XXM-S1	357.62 ± 37.1			
	C6-XXM-S2	287.37 ± 11.87			
Figure 4A	**OF task: distance (cm)**		**3Way-ANOVA followed by Tukey test**		
	B1-XYM-S1	4,379.025 ± 243.553	B1-XYM-S2 vs. C6-XYM-S2	*q* = 0.588	0.99
	B1-XYM-S2	2,774.775 ± 162.881	B1-XYF-S2 vs. C6-XYF-S2	*q* = 3.531	0.354
	C6-XYM-S1	4,471.654 ± 238.138	B1-XXM-S2 vs. C6-XXM-S2	*q* = 1.588	0.99
	C6-XYM-S2	2,656.923 ± 168.087			
	B1-XYF-S1	4,139.243 ± 290.059			
	B1-XYF-S2	3,025.794 ± 220.992			
	C6-XYF-S1	3,905.103 ± 139.724			
	C6-XYF-S2	2,342.452 ± 160.658			
	B1-XXM-S1	4,105.682 ± 196.187			
	B1-XXM-S2	3,143.498 ± 199.954			
	C6-XXM-S1	4,498.896 ± 209.671			
	C6-XXM-S2	3,114.608 ± 188.181			
Figure 4B	**Self-grooming Time (s)**		**2Way-ANOVA followed by Tukey test**		
	B1-XYM	10.384 ± 4.522	B1-XYM vs C6-XYM	*q =* 0.419	0.99
	C6-XYM	8.571 ± 3.557	B1-XYF vs. C6-XYF	*q =* 1.755	0.814
	B1-XYF	16.908 ± 6.947	B1-XXM vs. C6-XXM	*q =* 0.557	0.99
	C6-XYF	9.596 ± 2.884			
	B1-XXM	13.332 ± 3.282			
	C6-XXM	11.148 ± 3.664			
Figure 4C	**Marbles buried (#)**		**2Way-ANOVA followed by Tukey test**		
	B1-XYM	9.083 ± 1.104	B1-XYM vs. C6-XYM	*q =* 0.321	0.99
	C6-XYM	9.571 ± 2.202	B1-XYF vs. C6-XYF	*q =* 0.476	0.99
	B1-XYF	5.714 ± 1.392	B1-XXM vs. C6-XXM	*q =* 0.719	0.99
	C6-XYF	5 ± 1.115			
	B1-XXM	13.071 ± 1.344			
	C6-XXM	12.167 ± 1.342			
Figure 5B	**Three-Chamber task: social approach (s)**		**3Way-ANOVA followed by Tukey test**		
	B1-XYM-ms	52.992 ± 6.291	B1-ms vs. C6-ms	*q =* 4.159	0.02
	B1-XYM-ob	28.381 ± 3.58	B1-XYM-ms vs. B1-XYM-ob	*q =* 4.332	0.104
	C6-XYM-ms	58.071 ± 9.94	C6-XYM-ms vs. C6-XYM-ob	*q =* 4.085	0.159
	C6-XYM-ob	30.339 ± 4.103	B1-XYF-ms vs. B1-XYF-ob	*q =* 8.578	1.169 × 10^−6^
	B1-XYF-ms	83.767 ± 11.282	C6-XYF-ms vs. C6-XYF-ob	*q =* 13.857	3.143 × 10^−9^
	B1-XYF-ob	25.529 ± 4.122	B1-XXM-ms vs. B1-XXM-ob	*q =* 10.212	2.832 × 10^−8^
	C6-XYF-ms	94.801 ± 6.046	C6-XXM-ms vs. C6-XXM-ob	*q =* 14.96	1.5 × 10^−15^
	C6-XYF-ob	25.763 ± 3.026			
	B1-XXM-ms	77.182 ± 4.65			
	B1-XXM-ob	28.156 ± 2.292			
	C6-XXM-ms	102.583 ± 9.709			
	C6-XXM-ob	25.011 ± 2.041			
Figure 5C	**Three-Chamber task: discrimination ratio**		**2Way-ANOVA followed by Tukey test**		
	B1-XYM	0.295 ± 0.075	B1-XYM vs. C6-XYM	*q =* 0.102	0.99
	C6-XYM	0.287 ± 0.066	B1-XYF vs. C6-XYF	*q =* 0.751	0.99
	B1-XYF	0.453 ± 0.049	B1-XXM vs. C6-XXM	*q =* 1.927	0.748
	C6-XYF	0.565 ± 0.073			
	B1-XXM	0.51 ± 0.079			
	C6-XXM	0.562 ± 0.049			

*Ab, antibody; CP, cortical plate; CZ, cortical zones (including the subplate, intermediate zone and ventricular zone); IZ, intermediate zone; ms, mouse-stimulus; ob, object; SP, subplate; S1, session 1; S2, session 2; VZ, ventricular zone*.

Importantly, the CP/CZ ratio of B1-XYM and B1-XYF offspring did not differ (Figure 2C, *P* > 0.1, Table 1) suggesting that sex hormones do not determine the CP size in XY mice. Also, B1-XXM and B1-XXF mice had an equivalent CP/CZ ratio (Figure 2C, *P* > 0.1, Table 1), suggesting again that sex hormones do not regulate cortical structure in XX mice.

Additionally, when compared to their respective control group, the CP area was significantly smaller in C6-XYM fetuses (Figure 2D, *P* < 0.05, Table 1). As in the case of the CP/CZ ratio, two-way ANOVA showed significant effects for antibody [*F*_(1, 41)_ = 6.74, *P* = 0.001] and antibody × genotype interaction [*F*_(3, 41)_ = 3.42, *P* = 0.027], whereas the Tukey test confirmed the C6 vs. B1 effect (*q* = 3.82, *P* = 0.001), a significant C6-XYM vs. B1-XYM interaction (*q* = 4.98, *P* = 0.024) and lack of significance for all other interactions (Supplementary Table 1). Moreover, *in utero* exposure to C6 maternal antibody did not alter the CZ area in any of the groups (Figure 2E, *P* > 0.1, Table 1), which was verified by two-way ANOVA followed by Tukey test (Supplementary Table 1). To further confirm this point, we performed separate measurements of the areas of the subplate, intermediate zone, and ventricular zone (Figure 2A) and, again, found no differences across the groups (*P* > 0.5, Supplementary Table 1). Hormones were not identified as a contributing factor to the male bias of a C6-induced decrease in CP area at the E15.5 stage of development because neither gonadal female nor gonadal male XX fetuses were significantly affected by antibody exposure.

### C6-Mediated ASD-Like Phenotypes Are Influenced by Sex Chromosome Complement

To evaluate the role of sex chromosomes and gonadal hormones on the male bias of C6-induced behavioral deficits, we conducted a series of behavioral studies on adult FCG mice that were exposed to C6 or B1 *in utero* on E13.5. We include 3 groups in these studies (XYM, XYF and XXM) as our previous studies in wildtype C57BL/6 mice revealed no effect of C6 exposure on female offspring and there was no structural phenotype associated with C6 exposure in XXF offspring (Figures 2B,C). Initially, we performed an observational screen and surveyed several variables to assess muscle, spinal, spinocerebellar, sensory, neuropsychiatric, and autonomic functions (Table 2). We found that, for each genotype, there were no significant differences in the scores obtained for C6 and B1 mice (Table 2).

**Table 2:** Five functions were evaluated in the observational screen.

**Function**	**Variables**			
Muscle and spinal function	Abdominal tone, body position, body tone, contact righting, defecation, gait, grip strength, limb grasping, limb tone, pelvic elevation, positional passivity, righting reflex, spontaneous activity, tail elevation, trunk curl, urination, visual placing, wire maneuver			
Spino-Cerebellar function	Abdominal tone, body position, body tone, contact righting, gait, grip strength, limb grasping, limb tone, pelvic elevation, righting reflex, tail elevation, trunk curl, visual placing			
Sensory function	corneal reflex, gait, negative geotaxis, pinna reflex, righting reflex, toe pinch, transfer arousal, visual placing			
Neuro-Psychiatric function	Aggressivity, body position, body tone, contact righting, fear (to human handler), irritability, latency to move, locomotion, negative geotaxis, positional passivity, righting reflex, spontaneous activity, startle response, transfer arousal, tremor, vocalizations, wire maneuver			
Autonomic function	Defecation, heart rate, lacrimation, palpebral closure, piloerection, respiratory rate, salivation, skin color, startle response, tail elevation, urination			
**Groups**	**Mean** **±** **SEM**	**Interactions**	**Statistic**	***P*** **-value**
**Muscle and spinal function**	**2Way-ANOVA followed by Tukey test**
B1-XYM	26.333 ± 0.414	Ab	*F_(1, 64)_* = 0.236	0.798
C6-XYM	25.571 ± 0.369	Genotype	*F_(2, 64)_* = 0.331	0.719
B1-XYF	26.642 ± 0.561	B1-XYM vs C6-XYM	*q =* 0.751	0.99
C6-XYF	25.857 ± 0.962	B1-XYF vs. C6-XYF	*q =* 1.055	0.97
B1-XXM	26.231 ± 0.482	B1-XXM vs. C6-XXM	*q =* 1.066	0.97
C6-XXM	26.083 ± 0.609			
**Spino-Cerebellar function**	**2Way-ANOVA followed by Tukey test**
B1-XYM	20.16667 ± 0.112	Ab	*F_(1, 64)_* = 0.716	0.4
C6-XYM	19.57143 ± 0.202	genotype	*F_(2, 64)_* = 1.399	0.255
B1-XYF	20.35714 ± 0.169	B1-XYM vs. C6-XYM	*q =* 0.551	0.99
C6-XYF	20.28571 ± 0.184	B1-XYF vs. C6-XYF	*q =* 3.097	0.257
B1-XXM	20.23077 ± 0.231	B1-XXM vs. C6-XXM	*q =* 1.999	0.718
C6-XXM	20 ± 0.213			
**Sensory function**	**2Way-ANOVA followed by Tukey test**
B1-XYM	11.625 ± 0.186	Ab	*F_(1, 64)_* = 0.17	0.681
C6-XYM	12.071 ± 0.229	Genotype	*F_(2, 64)_* = 1.161	0.32
B1-XYF	11.679 ± 0.219	B1-XYM vs. C6-XYM	*q =* 0.336	0.99
C6-XYF	11.714 ± 0.486	B1-XYF vs. C6-XYF	*q =* 0.125	0.99
B1-XXM	12.038 ± 0.215	B1-XXM vs. C6-XXM	*q =* 0.894	0.99
C6-XXM	11.875 ± 0.175			
**Neuro-Psychiatric function**	**2Way-ANOVA followed by Tukey test**
B1-XYM	38.333 ± 1.558	Ab	*F_(1, 64)_* = 0.681	0.412
C6-XYM	31.714 ± 1.714	Genotype	*F_(2, 64)_* = 0.64	0.52
B1-XYF	40.143 ± 1.123	B1-XYM vs. C6-XYM	*q =* 0.122	0.99
C6-XYF	38.142 ± 1.404	B1-XYF vs. C6-XYF	*q =* 1.93	0.747
B1-XXM	34.692 ± 1.184	B1-XXM vs. C6-XXM	*q =* 0.147	0.99
C6-XXM	40.333 ± 1.514			
**Autonomic function**	**2Way-ANOVA followed by Tukey test**
B1-XYM	7.75 ± 0.25	Ab	*F_(1, 64)_* = 0.689	0.409
C6-XYM	7.857 ± 0.634	Genotype	*F_(2, 64)_* = 1.495	0.232
B1-XYF	8 ± 0.392	B1-XYM vs. C6-XYM	*q =* 2.033	0.707
C6-XYF	6.857 ± 0.404	B1-XYF vs. C6-XYF	*q =* 0.508	0.99
B1-XXM	8.077 ± 0.309	B1-XXM vs. C6-XXM	*q =* 0.458	0.99
C6-XXM	7.833 ± 0.441			

*Variables in the second column (assessing multiple aspects of each function) were considered, with their individual scores being added per function. The statistical analysis of the functions from the observational screen showed that in utero exposure to C6 did not significantly alter the five functions. The number of mice per group was: B1-XYM = 12, C6-XYM = 7, B1-XYF = 7, C6-XYF = 13, B1- XXM = 14, and C6-XXM = 12. Two-way ANOVA with Tukey correction, which did not reach significance (P <0.05); Ab, antibody*.

**Figure 3:**
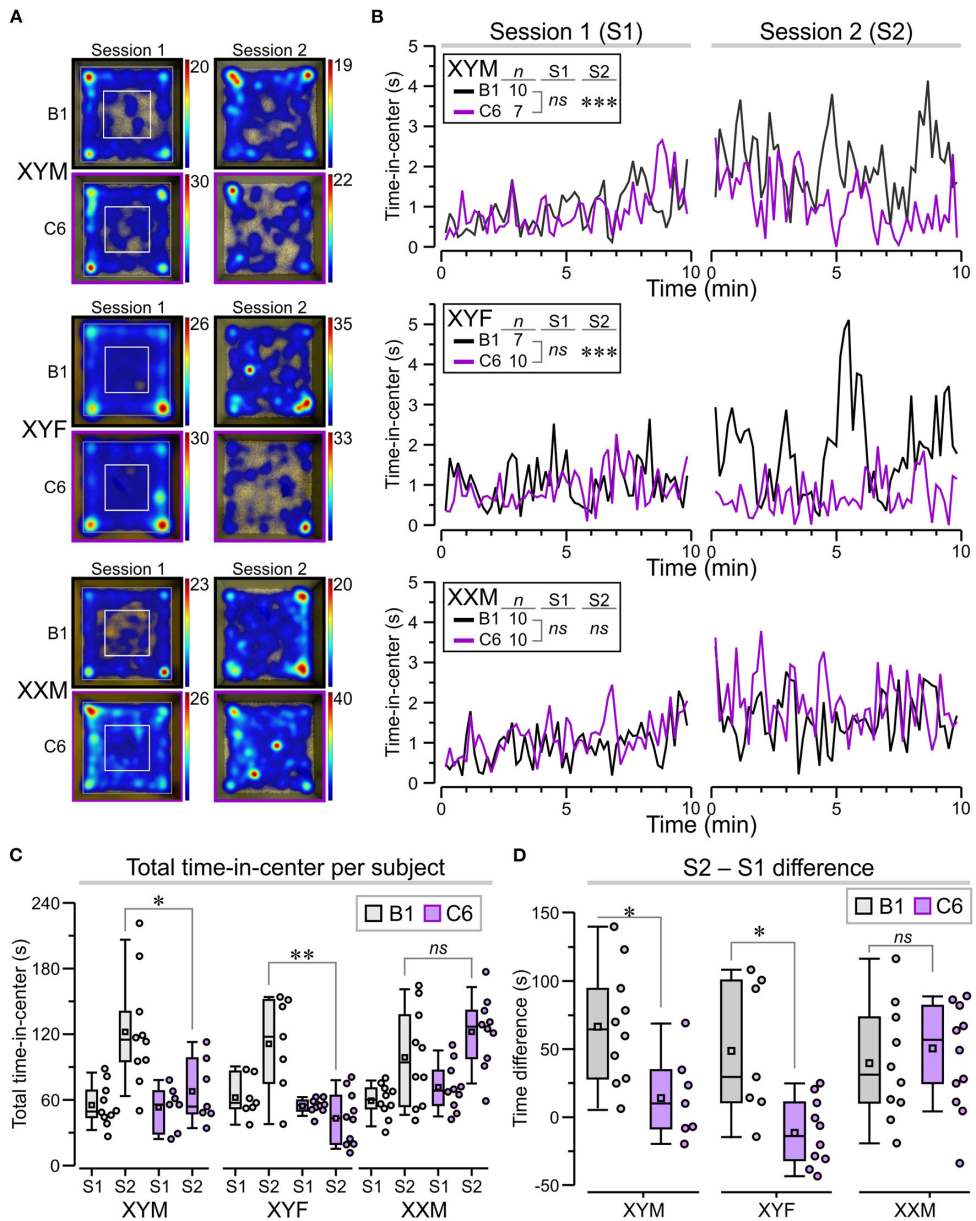
Sustained anxiety-like behavior in C6-exposed XYM and XYF mice during the open-field task. **(A)** Top-view heatmaps of the open-field task for representative XYM, XYF, and XXM mice that were exposed *in utero* to anti-Caspr2 antibody (C6) or control antibody (B1). The color scale at the right of each heatmap represents seconds. **(B)** Graphs show the time spent at the center of the arena, taken over regular 10-s intervals during session 1 (S1) and session 2 (S2), for all groups. RMANOVA with Bonferroni *post-hoc* tests reveal significantly lower time-in-center, during S2, for C6-XYM mice compared to B1-XYM mice as well as C6-XYF mice compared to B1-XYF mice. **(C)** Box-and-whisker plots for time-in-center show mean (small square), median, Q1–Q3 quartiles (box), and 10–90 range (whiskers) for S1 and S2. Dots represent individual mice; 3-way ANOVA, followed by Tukey test, was used for statistical comparisons. **(D)** Difference for time-in-center between S2 and S1 show statistical differences for the XYM and XYF cohorts; 2-way ANOVA with Tukey test was used for statistical comparisons. See Table 1 and Supplementary Table 2 for details of statistical testing; *ns* = non-significant, *P < 0.05, **P < 0.01, ***P < 0.001.

A set of behavioral assessments was focused on key ASD-like phenotypes, such as the presence of anxiety, repetitive behaviors, and social impairments. We studied adult mice from eight litters exposed to C6 and six litters exposed to B1. Not all the genotypes were represented in each litter. Anxiety is often a comorbid psychiatric condition in patients with ASD. Therefore, we evaluated C6-exposed FCG mice for increased anxiety-like behavior in an open-field task in which the animals were allowed to freely explore a well-lit square arena during two sessions (S1 and S2), lasting 10 min each, and separated by 24 h (Figure 3A). In the context of the open-field task, anxiety-like behavior is defined as avoidance to explore the center of the apparatus, which some claim occurs more prominently at the early stage of the task. However, it is clear that initial exposure to the chamber triggers habituation, a type of non-associative learning, in which the mice adapt their behavioral responses due to the continual exposure to the novel environment. Therefore, in this study, we decided to define sustained anxiety-like behavior as a mouse spending significantly less time in the center of the arena during S2, after the initial S1 habituation session. We, thus, calculated ‘time-in-center' scores for consecutive 10-s intervals in S1 and S2, which we used to build time series graphs (Figure 3B). During S1, we found that all groups (regardless of antibody exposure or genotype) displayed similar time-in-center scores (Figure 3B, *P* > 0.1, Table 1). During S2, C6-XYM mice and C6-XYF mice did not increase their time-in-center scores while the other groups spent significantly more time in the center area than they had during S1 (Figure 3B, *P* < 0.01, Table 1, Supplementary Table 2).

To further demonstrate the anxiety-like effect in C6-XYM and C6-XYF groups, we computed the overall time-in-center for each subject during S1 and S2 (Figure 3C) and found that neither C6-XYM nor C6-XYF mice increased the amount of time spent in the center during S2 (*P* = 0.99 for both groups, Table 1, Supplementary Table 2). In contrast, C6-XXM mice significantly increased their time-in-center during S2 (*P* < 0.05, Table 1, Supplementary Table 2). Also, B1-XYM mice had higher time-in-center during S2 (Figure 3C, *P* < 0.05, Table 1, Supplementary Table 2). Furthermore, a comparison of B1 vs C6 groups during S2 confirmed that C6-XYM and C6-XYF mice spent significantly less time-in-center than their B1 counterparts (Figure 3C, *P* < 0.05 for both groups, Table 1, Supplementary Table 2), whereas C6-XXM and B1-XXM animals spent a similar amount time in the center of the arena (Figure 3C, *P* = 0.796, Table 1, Supplementary Table 2). Finally, we computed the time difference for time-in-center during the two sessions (S2–S1) and found that the C6-XYM and C6-XYF groups had significantly lower scores than B1-XYM and B1-XYF groups, respectively (Figure 3D, *P* < 0.05 for both groups, Table 1, Supplementary Table 2). Taken together, these data show a sustained anxiety-like phenotype associated with sex chromosome complement.

**Figure 4:**
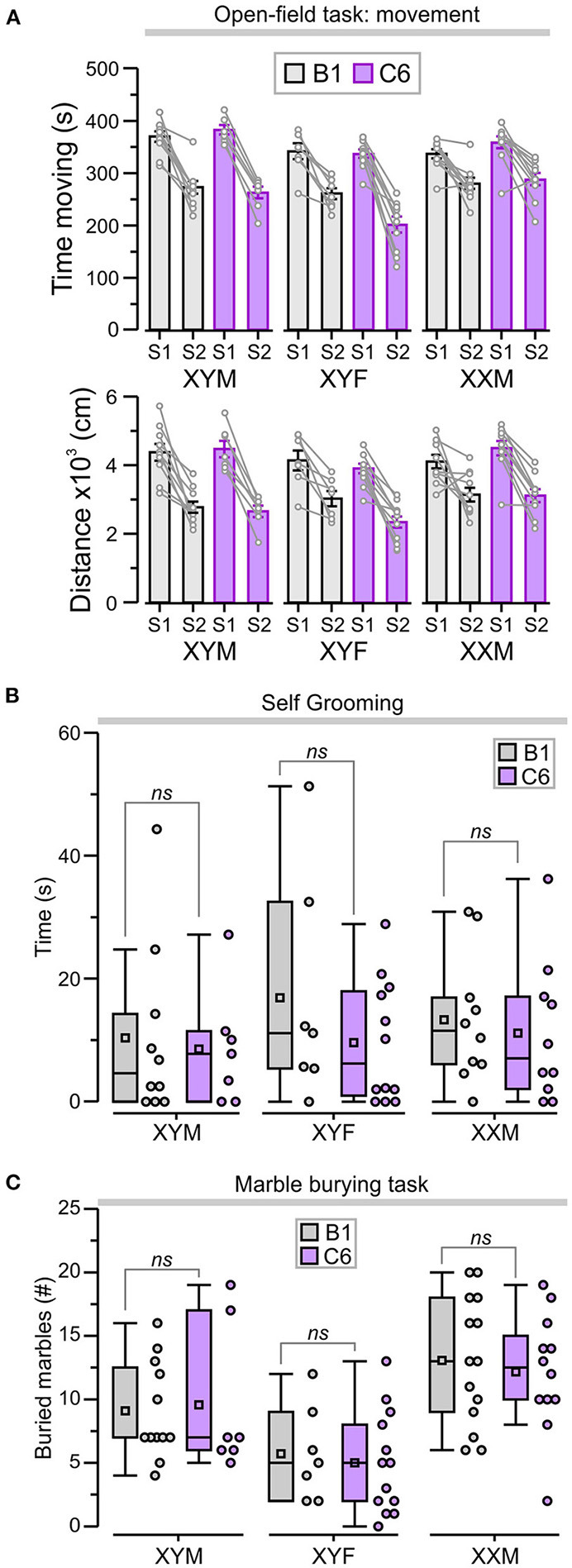
C6 exposure *in utero* did not lead to an increase in repetitive behaviors. **(A)** *Top*, graph shows the total time (mean ± SEM) moving during sessions 1 and 2 (S1 and S2). *Bottom*, total distance traveled (mean ± SEM) as a measure of locomotor activity in the open-field task. The lines linking each S1 and S2 represent the scores for individual animals. **(B)** Box-and-whisker plots for cumulative time self-grooming show median and Q1–Q3 quartiles (whiskers are 10–90 range). Dots represent individual animals. C6-exposed mice did not exhibit increased self-grooming compared to B1 control groups, irrespective of genotype. **(C)** Box-and-whisker plots show the number of marbles buried (more than 50% of the surface area covered by bedding) during the marble-burying task. The number of marbles buried was not significantly different between B1- and C6-exposed mice, irrespective of genotype; 2-way ANOVA with Tukey test was used for statistical comparisons. See Table 1 and Supplementary Table 2 for details of statistical testing; *ns* = non-significant.

We also assessed locomotor activity in the open-field task by measuring the total time moving and the total distance traveled during each period to ensure that this was not a confounding variable. We focused on S2 as it was in S2 that we observed differences related to genotype and antibody exposure. During S2, all the groups showed significantly less time moving when compared to S1 (Figure 4A, *P* < 0.05, Table 1, Supplementary Table 2); nevertheless, the C6-exposed offspring had similar time-moving scores when compared to B1 control mice (Figure 4A, *P* > 0.5, Table 1, Supplementary Table 2). Additionally, there were no significant differences in distance traveled during S2 for C6-exposed mice compared to their respective B1-exposed controls (Figure 4A, *P* > 0.05, Table 1, Supplementary Table 2).

We evaluated the C6-exposed FCG mice for stereotypic and compulsive behaviors using cumulative time self-grooming and number of marbles buried. We found that XYM, XYF, and XXM mice exposed to C6 *in utero* did not display an increase in repetitive behaviors when compared to their respective B1 controls in either self-grooming (Figure 4B, *P* > 0.4, Table 1, Supplementary Table 2) or the marble burying task (Figure 4C, *P* > 0.6, Table 1, Supplementary Table 2).

**Figure 5:**
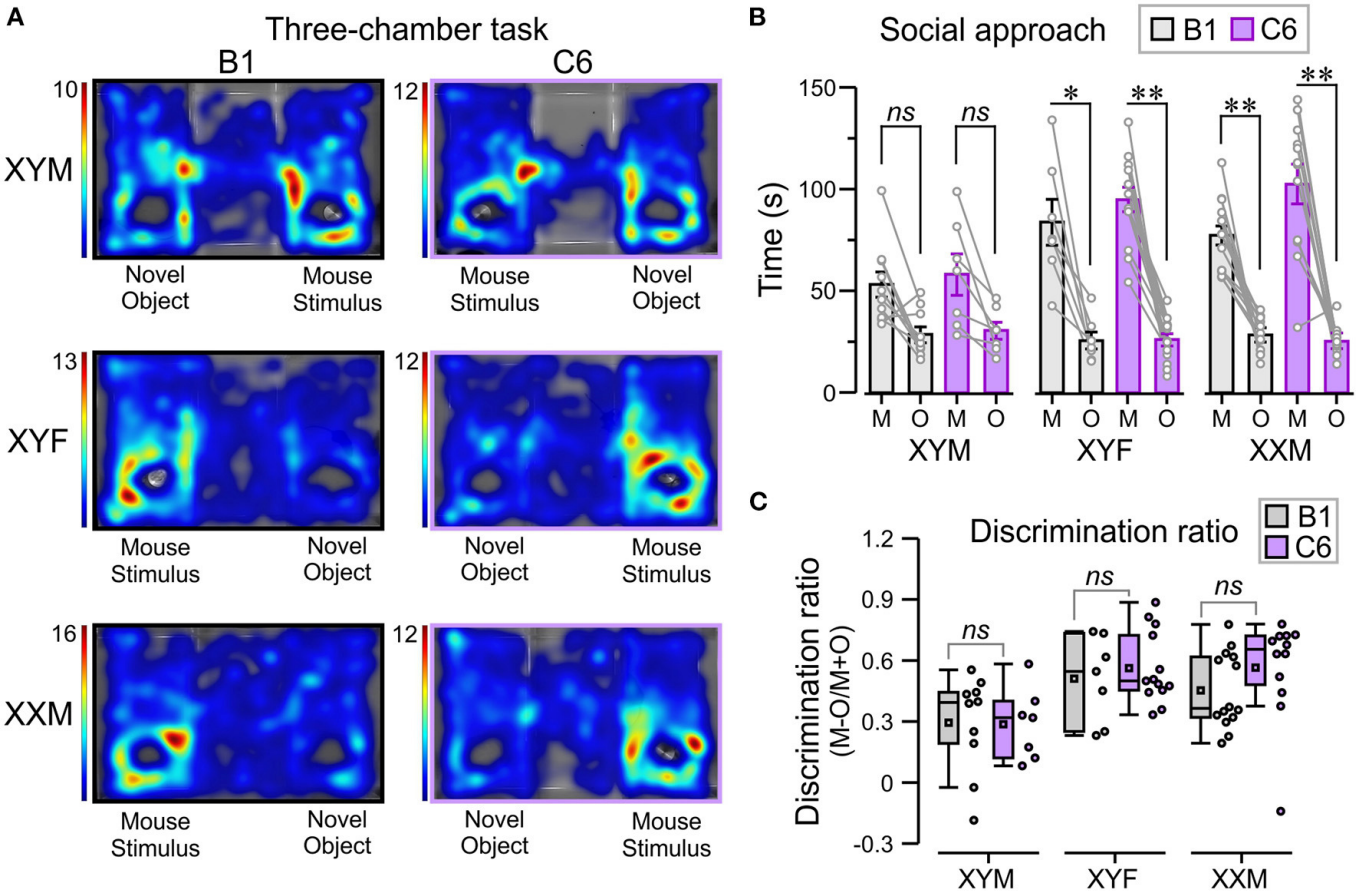
*In utero* C6 exposure did not affect social approach. We used the three-chamber task to evaluate social approach. The number of mice per group was: B1-XYM = 10, C6-XYM = 7, B1-XYF = 7, C6-XYF = 13, B1-XXM = 14, and C6-XXM = 12. **(A)** Representative trials of the social approach session displayed as top-view heatmaps for individual B1- and C6-exposed XYM, XYF, and XXM mice. **(B)** Time (mean ± SEM) spent interacting with the novel object (O) and the “mouse-stimulus” (M). The lines linking each mouse-stimulus and object represent the scores for individual subjects. All the groups tested showed social preference for the mouse-stimulus compared to the novel object; 3-way ANOVA with Tukey test was used for these comparisons. **(C)** Box-and-whisker plots show the discrimination ratio for social interaction. C6- and B1-exposed mice displayed similar discrimination between mouse-stimulus and novel object, irrespective of genotype; 2-way ANOVA with Tukey test was used for these comparisons. See Table 1 and Supplementary Table 2 for details of statistical testing; *ns*, non-significant, *P < 0.05, **P < 0.001.

Lastly, we used the three-chamber task [adapted from Yang et al. (26)] to explore the influence of sex chromosomes and gonadal hormones on the decreased social interactions produced by *in utero* exposure to C6 in male mice (7) (Figure 5A). We defined social approach as the total amount of time spent interacting with an unfamiliar mouse (referred to as “mouse-stimulus”) compared to the amount of time spent interacting with a novel object. Normal social approach was defined as spending more time near the mouse-stimulus and less time near the object. We found that XYF and XXM mice, regardless of C6 or B1 exposure, preferred the mouse-stimulus compared to the object (Figures 5A,B, *P* < 0.01, Table 1, Supplementary Table 2). Interestingly, C6-XYM mice did not show significantly higher exploration of the mouse-stimulus but, paradoxically, B1-XYM mice failed to show a preference for the mouse-stimulus (Figures 5A,B, *P* > 0.1, Table 1, Supplementary Table 2). Additionally, we computed social discrimination ratios (for which positive values reflect a predilection for the mouse-stimulus), which further demonstrated that B1- and C6-exposed mice of all groups preferred the mouse-stimulus instead of the object (Figure 5C, Table 1, Supplementary Table 2). These results indicate that XYM, XYF and XXM mice exposed to C6 displayed normal social approach. We were unable to replicate the phenotype seen in C6 exposed male wild type mice (7) in the FCG model.

## Discussion

In this study we used the FCG mouse model to distinguish the contributions of sex chromosomes and gonadal hormones to abnormal cortical development and adult ASD-like phenotypes in a model of maternally-induced ASD by the anti-Caspr2 antibody, C6. Analysis of the fetal brains showed that C6-exposed XYM fetuses had significantly smaller CP/CZ ratio and CP area, when compared to B1-XYM control fetuses. Remarkably, the B1-XYM and B1-XYF fetal cortices did not differ in their CP/CZ ratio and CP area, and this was also the case for the B1-XXM and B1-XXF groups, which strongly suggest that sex hormones were not responsible for determining the CP size in either XY or XX mice. Moreover, behavioral assessment of adult offspring revealed a behavioral pattern of sustained anxiety-like behavior in C6-XYM and C6-YXF mice, in which they spent significantly less time in the center of an open-field arena during a second exposure after the initial habituation session. Additionally, adult C6-exposed XYM, XYF and XXM mice did not seem to exhibit abnormalities in locomotion, self-grooming, the marble-burying task and the three-chamber social approach task.

C6 antibody exposure in our model occurs throughout a limited window during *in utero* development, from time of antibody administration on E13.5 to IgG exclusion by the blood brain barrier between E16.5 and E17.5. Thus, we propose that the sex-biased effects of the C6 antibody depend on the roles that sex chromosome genes and gonadal hormones have on the critical brain developmental processes ongoing at the time of exposure. Our findings support a predominant influence of sex chromosome complement on the susceptibility to C6-induced ASD-like phenotypes in mice exposed mid-gestation.

Changes in cortical thickness, including thinning (27–32) and thickening (33–35), have been observed in ASD. We have previously shown that *in utero* exposure to C6 antibody leads to thinning of the CP in male mice (7). Using the FCG mouse model, we found that both XYM and XYF but not XXM or XXF mice had a significantly smaller CP/CZ ratio when exposed to C6. Thus, presence of the Y chromosome or lack of two copies of the X chromosome is key in determining the susceptibility to cortical thinning, irrespective of gonadal hormones. The mechanism for the thinned cortical plate has not yet been determined. Sex chromosome complement has previously been identified to be important for brain development and the establishment of brain sex differences (21, 36–40). In congruence with our findings, the analysis of MR brain images from individuals with complete androgen insensitivity syndrome (CAIS) and Klinefelter syndrome suggests a sex chromosome gene-dosage effect on the thickness of the motor cortex (39) and the temporal, orbitofrontal and lingual cortices (41), respectively. Savic and Arver (41) propose that sex differences in motor cortex development are predominantly established by X-chromosome genes that escape inactivation and lack a Y-chromosome homolog whereas in the superior temporal cortex these differences are likely influenced by X-chromosome escapee genes with Y-chromosome homologs. Similar potential sex chromosome gene dosage relationships have been proposed to account for the brain structural differences identified in individuals with sex chromosome aneuploidies (42–44). Indeed, Vawter et al. (45) identified fourteen X-chromosome genes that are differentially expressed in XXY compared to XY individuals, twelve of which were significantly correlated with measure of verbal cognition. Furthermore, Good et al. (42) propose that haploinsufficiency of a subset of X-linked genes, including the gene encoding monoamine oxidase B, contribute to the brain structural abnormalities and neurocognitive deficits observed in Turner syndrome. The increased prevalence of ASD in individuals with sex chromosome aneuploidies [reviewed in (46–51)] supports the hypothesis that sex chromosome gene dosage contributes to ASD susceptibility and suggests that it may contribute to the sex bias in our model.

While sex chromosomes are essential for brain differentiation and sex specific behaviors (21, 37, 52–59), gonadal hormones also modulate developmental processes and contribute to sex differences in brain anatomy. Indeed, both human and animal studies have found a mixed contribution of gonadal hormones and sex chromosomes to sex differences in brain structure, with the predominant factor being region specific (38, 39, 41, 43, 6041; 39). In particular, testosterone exposure has been implicated in determining parietal and occipital cortical thickness in individuals with CAIS and sex chromosome aneuploidies (39, 41). Additionally, data from the FCG mouse model also suggest a predominant gonadal hormone contribution to differences in cortical thickness and volume in the adult brain as we found in the comparison of B1-exposed XXF and XXM mice in the fetal brain (38, 60). Based on these data, gonadal hormones might have been predicted to modulate the effects of C6 on fetal cortical thickness, however, they mainly address hormone exposure during puberty (61, 62). Furthermore, gonadal hormone and signaling deficiencies observed in individuals with CAIS and sex chromosome aneuploidies may act as confounders when studying the association between sex chromosomes and brain sexual differentiation.

While sex hormones act on the brain throughout post-partum life, for gonadal hormones to modulate the susceptibility to C6-induced phenotypes, both the hormones and their receptors must be expressed at the time of antibody exposure. Androgen receptor (AR) mRNA has been detected in the fetal mouse brain as early as E11, with expression in the neocortex, hippocampal cortex, and hypothalamus peaking on E15–16 (63). Estrogen receptor (ER) α protein has been detected as early as E12–14 in the mouse embryonic ventricular and subventricular zones (64) whereas mRNA has been detected as early as E16.5 in the mouse fetal brain (65) and follows a similar pattern in the ventricular zone and cerebral cortex in rats, with the earliest expression on E16 (66). ERβ protein has been detected as early as E12.5 in the mouse brain and was first appreciated in the deep layers of the cerebral cortex on E15.5–16.5 (67). Signaling through AR and ERs has been found to regulate developmental processes in the cortex. Zhang et al. (68) observed an effect of testosterone and estrogen on the differentiation but not proliferation of cortical neurons isolated from rat fetuses on E14. They proposed that these effects are mediated by signaling through the AR and not ERα as its expression was low. In mice, Wang et al. (69) proposed that estrogen regulates neuronal survival and migration through ERβ and that decreased signaling leads to cortical thinning likely secondary to impairments in these processes. While the beginning of the period during which decreased signaling through ERβ in the mouse fetal brain leads to cortical thinning was narrowed down to E14.5 at the earliest, thus overlapping with the period of antibody exposure in our model, no sex bias was observed. This lack of sex bias in the effects of altered signaling through ERβ on cortical structure could account for the insignificant influence of gonadal hormones in our ASD model as one would expect C6 to interact with processes that when altered have different outcomes in males compared to females.

Both testosterone and estrogens are present in mouse fetal circulation at the time of C6 exposure. Fetal Leydig cells in mice arise on E12.5 [reviewed in (70)] and proteins required for testosterone synthesis are first detected between E12.5 and E13.5, coinciding with the earliest reported testosterone production (71). Given that fetal rodent ovaries are thought to produce minimal amounts of estrogen (72), estrogens in the fetal peripheral circulation likely originate from maternal and placental sources. However, of these hormones, only peripherally derived testosterone crosses the blood brain barrier in a form that can be actively used. Estrogens in the peripheral circulation are bound by alpha-fetoprotein, rendering them inaccessible for cell signaling in the brain (73). Thus, alpha-fetoprotein protects the female rodent brain from masculinization and defeminization by estrogen (73) while testosterone aromatization to estrogen masculinizes and defeminizes the male rodent brain. While alpha-fetoprotein may sequester peripheral estrogens, other sources of estrogen including aromatization of testosterone and *de novo* synthesis from cholesterol have been identified in the developing rodent brain (74–83). Moreover, Martínez-Cerdeño et al. (64) detected aromatase expression in the mouse ventricular and subventricular zones as early as E9. Interestingly, alpha-fetoprotein is also strongly expressed in the ventricular zone from E12 (64). Hence, while there may be differences in the level of estrogen in the brain during development between males and females, C6 may alter brain developmental processes in regions where estrogen signaling is diminished by locally produced alpha-fetoprotein. Furthermore, sex chromosome genes may modulate aromatase and gonadal hormone receptor expression in the brain (84, 85), potentially accounting for the observed dominant genetic effect on C6 susceptibility. In accordance with this hypothesis, decreased expression of aromatase has been detected in individuals with ASD (86, 87).

The behavioral assessment of adult mice revealed that the time spent in the center of an open-field arena did not significantly increase upon repeat exposure in C6-XYM and C6-XYF mice. Furthermore, C6-XYM and C6-XYF mice spent significantly less time in the center of the arena during repeat exposure compared to the control B1 counterparts. We have termed this response sustained anxiety-like behavior, as anxiety has been defined by others as avoidance of the center of an arena in the first exposure. Together, these observations indicate an abnormal behavioral pattern due to *in utero* C6 exposure in XYM and XYF mice. Conversely, XXM mice showed a significant difference in time spent in the center between the first and repeated exposures independent of C6 or B1 exposure, suggesting that the anxiety phenotypes are driven by sex chromosome complement. Indeed, individuals with sex chromosome aneuploidies, including Klinefelter and Turner Syndromes, have been reported to have an increased prevalence of anxiety (88–90). Additional tests including the elevated plus maze and light-dark box tests may be useful to further characterize the anxiogenic effects of C6 and the relationship to sex chromosome complement.

In this study we identified sex chromosome complement to be essential for the effects of *in utero* exposure to C6 maternal antibody on fetal brain cortical development and adult behavior. Differences in gene dosage is one potential mechanism through which sex chromosome complement may be increasing the susceptibility of XY mice to C6. Indeed, Xu et al. (59) identified a subset of genes outside the pseudo-autosomal region of the sex chromosomes whose level of expression in the brain shows a sex bias. Furthermore, while X chromosome inactivation is a compensatory mechanism for differences in gene dosage between XX and XY complements (91), an estimated 10–15% of the X chromosome genes outside the pseudo-autosomal region escape inactivation in humans and are therefore more highly expressed in XX compared to XY individuals (92, 93). Finally, imprinting can affect expression levels of X chromosome genes in the brain (94–96) and has been associated with impairments in social behavior in Turner Syndrome (97) and cognitive function in a mouse model of this condition (94). Of note, the mouse Y chromosome encodes for 10 × more genes than the human Y chromosome (98). Consequently, if Y chromosome genes account for the male bias observed in our mouse model of maternal antibody induced ASD, our findings may not completely translate to the pathogenesis of ASD in humans exposed *in utero* to anti-Caspr2 antibody.

While the establishment of sexual dimorphisms involves both gonadal hormones and sex chromosomes, both of which are operative not just during the window of fetal brain exposure to maternal antibody our data suggest that gonadal hormones have a limited role in determining the susceptibility to C6-induced phenotypes, at least in these genetically manipulated mice. It should be noted that sex hormone levels in these mice are not the same as in the C57BL/6 strain in which the C6 model was established. Nevertheless, C6 likely impairs development processes that are regulated by sex chromosome genes. It is possible that these sex chromosome genes modulate gonadal hormone signaling pathways. For example, sex chromosome complement determines sex differences in aromatase and ERβ expression levels in the developing mouse amygdala (84, 85). Given the proposed relationship between cortical thickness and sociability (30) and symptom severity (99) in ASD, further inquiry into the exact mechanism by which sex chromosome complement influences the risk to develop ASD-like phenotypes due to C6 exposure *in utero* is important. Deciphering the exact mechanisms through which sex chromosome genes compensate for or exacerbate the effects of *in utero* C6 exposure in females and males, respectively, will be key for expanding our knowledge of brain development and identifying potential therapeutic targets for ASD.

## Data Availability Statement

The original contributions presented in the study are included in the article/Supplementary Material, further inquiries can be directed to the corresponding author/s.

## Ethics Statement

The animal study was reviewed and approved by Institutional Animal Care and Use Committee (IACUC) of the Feinstein Institutes for Medical Research.

## Author Contributions

AG-G, AP, LB, BV, PH, and BD designed the experiments. AG-G, AP, BV, and PH performed experiments and analyzed the data. AG-G and PH made the final figures. AG-G, PH, and BD wrote the manuscript. All authors approved the manuscript.

## Funding

This work was supported by the National Institutes of Health (NIH) grant 5P01AI102852 and NIH grant 5P01AI073693 to BD. It was also supported by the Nancy Lurie Marks Family Foundation (BD). PH was supported by DOD Impact Award W81XWH1910759.

## Conflict of Interest

The authors declare that the research was conducted in the absence of any commercial or financial relationships that could be construed as a potential conflict of interest.

## Publisher's Note

All claims expressed in this article are solely those of the authors and do not necessarily represent those of their affiliated organizations, or those of the publisher, the editors and the reviewers. Any product that may be evaluated in this article, or claim that may be made by its manufacturer, is not guaranteed or endorsed by the publisher.
