# Supplementary material for: Contributions of Sex Chromosomes and Gonadal Hormones to the Male Bias in a Maternal Antibody-Induced Model of Autism Spectrum Disorder
Source: Front Neurol. 2021 Oct 13;12:721108. doi: 10.3389/fneur.2021.721108 (PMC8548617; doi:10.3389/fneur.2021.721108)
Supplement: Supplementary file 1 [file Data_Sheet_1.PDF]

Supplementary Material

SUPPLEMENTAL TABLE 1: Statistical analysis for the fetal brain study. Two-way ANOVA, with 'genotype' (XYM, XXM, XYF and XXF) and 'antibody' (C6 and B1) as factors, was followed by post-hoc Tukey correction for multiple comparisons. Significance (Sig): Sig = 0 indicates that the difference of the means is not significant at the 0.05 level; Sig = 1 indicates that the difference of the means is significant at the 0.05 level. Abbreviations: Ab, antibody; CP, cortical plate; Gen, genotype; IZ, intermediate zone; SZ, cortical zones (including subplate, intermediate zone and ventricular zone); SP, subplate; and VZ, ventricular zone.

**Variable**	**Test / Factors**	**Statistic**	**P value**			**Statistic**	**P value**	
**CP/CZ ratio**	**2Way-ANOVA**	**F value**	**P**	**Sig**	**Interactions (cont.)**	**q value**	**P**	**Sig**
**(Fig. 2C)**	Ab	$F_{(1,41)}$ = 9.101	0.00481	1	C6-XYM vs. B1-XXM	2.82531	0.49803	0
	Genotype	$F_{(3,41)}$ = 0.868	0.46729	0	C6-XYM vs. B1-XXF	0.24165	0.99999	0
	Interaction	$F_{(3,41)}$ = 3.288	0.03229	1	C6-XYF vs. B1-XYM	4.47251	0.05807	0
	**Tukey Test**	**q value**			C6-XYF vs. B1-XYF	3.56502	0.22116	0
	Ab: B1 vs. C6	4.409	0.00369	1	C6-XYF vs. B1-XXM	2.55873	0.61838	0
	Gen: XYF vs. XYM	0.07059	0.99995	0	C6-XYF vs. B1-XXF	0.16673	0.99999	0
	Gen: XXM vs. XYM	0.05633	0.99998	0	C6-XYF vs. C6-XYM	0.06157	0.99999	0
	Gen: XXM vs. XYF	0.12151	0.99977	0	C6-XXM vs. B1-XYM	3.29438	0.30779	0
	Gen: XXF vs. XYM	1.64757	0.65254	0	C6-XXM vs. B1-XYF	2.38689	0.69467	0
	Gen: XXF vs. XYF	1.50985	0.71121	0	C6-XXM vs. B1-XXM	1.3806	0.97484	0
	Gen: XXF vs. XXM	1.63136	0.65954	0	C6-XXM vs. B1-XXF	1.0114	0.99594	0
	**Interactions**	**q value**			C6-XXM vs. C6-XYM	1.33409	0.9792	0
	B1-XYF vs. B1-XYM	0.90749	0.99793	0	C6-XXM vs. C6-XYF	1.17813	0.98981	0
	B1-XXM vs. B1-XYM	1.91378	0.87136	0	C6-XXF vs. B1-XYM	3.20947	0.33883	0
	B1-XXM vs. B1-XYF	1.00629	0.99606	0	C6-XXF vs. B1-XYF	2.30198	0.7308	0
	B1-XXF vs. B1-XYM	4.30579	0.07595	0	C6-XXF vs. B1-XXM	1.29568	0.98236	0
	B1-XXF vs. B1-XYF	3.39829	0.27227	0	C6-XXF vs. B1-XXF	1.09632	0.99336	0
	B1-XXF vs. B1-XXM	2.392	0.69245	0	C6-XXF vs. C6-XYM	1.42581	0.97	0
	C6-XYM vs. B1-XYM	4.89244	0.02847	1	C6-XXF vs. C6-XYF	1.26304	0.98474	0
	C6-XYM vs. B1-XYF	3.91223	0.13773	0	C6-XXF vs. C6-XXM	0.08491	0.99999	0
**CP area**	**2Way-ANOVA**	**F value**	**P value**	**Sig**	**Interactions (cont.)**	**q value**	**P value**	**Sig**
**(Fig.2D)**	Ab	$F_{(1,41)}$ = 6.74034	0.01382	1	C6-XYM vs. B1-XXM	2.77797	0.51917	0
	Genotype	$F_{(3,41)}$ = 1.10263	0.36149	0	C6-XYM vs. B1-XXF	0.05401	0.99999	0
	Interaction	$F_{(3,41)}$ = 3.42347	0.02797	1	C6-XYF vs. B1-XYM	4.55257	0.05089	0
	**Tukey Test**	**q value**			C6-XYF vs. B1-XYF	2.7406	0.53598	0
	Ab: B1 vs. C6	3.81741	0.01075	1	C6-XYF vs. B1-XXM	2.50788	0.64126	0
	Gen: XYF vs. XYM	0.72267	0.9559	0	C6-XYF vs. B1-XXF	0.11403	0.99999	0
	Gen: XXM vs. XYM	0.04327	0.99999	0	C6-XYF vs. C6-XYM	0.06916	0.99999	0
	Gen: XXM vs. XYF	0.73333	0.95407	0	C6-XXM vs. B1-XYM	3.28275	0.31194	0
	Gen: XXF vs. XYM	1.89276	0.54561	0	C6-XXM vs. B1-XYF	1.47078	0.96455	0
	Gen: XXF vs. XYF	1.12027	0.8575	0	C6-XXM vs. B1-XXM	1.23805	0.9864	0
	Gen: XXF vs. XXM	1.85361	0.56266	0	C6-XXM vs. B1-XXF	1.38385	0.97451	0
	**Interactions**				C6-XXM vs. C6-XYM	1.44072	0.96826	0
	B1-XYF vs. B1-XYM	1.81197	0.8995	0	C6-XXM vs. C6-XYF	1.26982	0.98427	0
	B1-XXM vs. B1-XYM	2.0447	0.82941	0	C6-XXF vs. B1-XYM	3.28224	0.31212	0
	B1-XXM vs. B1-XYF	0.23273	0.99999	0	C6-XXF vs. B1-XYF	1.47027	0.96461	0
	B1-XXF vs. B1-XYM	3.66661	0.42032	0	C6-XXF vs. B1-XXM	1.23754	0.98643	0
	B1-XXF vs. B1-XYF	2.85464	0.48504	0	C6-XXF vs. B1-XXF	1.38437	0.97446	0
	B1-XXF vs. B1-XXM	2.62191	0.58976	0	C6-XXF vs. C6-XYM	1.44128	0.9682	0
	C6-XYM vs. B1-XYM	4.9865	0.02411	1	C6-XXF vs. C6-XYF	1.27033	0.98423	0
	C6-XYM vs. B1-XYF	3.02935	0.41012	0	C6-XXF vs. C6-XXM	5.14237E-4	0.99999	0
**CZ area**	**2Way-ANOVA**	**F value**	**P value**	**Sig**	**Interactions (cont.)**	**q value**	**P value**	**Sig**
**(Fig.2E)**	Ab	$F_{(1,41)}$ = 3.56957	0.0674	0	C6-XYM vs. B1-XXM	2.50527	0.64243	0
	Genotype	$F_{(3,41)}$ = 0.95485	0.42516	0	C6-XYM vs. B1-XXF	0.06307	0.99999	0
	Interaction	$F_{(3,41)}$ = 2.54508	0.07234	0	C6-XYF vs. B1-XYM	3.97597	0.12557	0
	**Tukey Test**	**q value**			C6-XYF vs. B1-XYF	1.89934	0.8756	0
	Ab: B1 vs. C6	2.80715	0.05527	0	C6-XYF vs. B1-XXM	2.45661	0.66411	0
	Gen: XYF vs. XYM	1.16248	0.84366	0	C6-XYF vs. B1-XXF	0.07879	0.99999	0
	Gen: XXM vs. XYM	0.39143	0.99246	0	C6-XYF vs. C6-XYM	0.14817	0.99999	0
	Gen: XXM vs. XYF	1.48776	0.7204	0	C6-XXM vs. B1-XYM	2.42923	0.67619	0
	Gen: XXF vs. XYM	1.48776	0.75027	0	C6-XXM vs. B1-XYF	0.3526	0.99999	0
	Gen: XXF vs. XYF	0.24134	0.9982	0	C6-XXM vs. B1-XXM	0.90987	0.9979	0
	Gen: XXF vs. XXM	1.7291	0.61707	0	C6-XXM vs. B1-XXF	1.46795	0.96491	0
	**Interactions**				C6-XXM vs. C6-XYM	1.5225	0.95745	0
	B1-XYF vs. B1-XYM	2.07663	0.81826	0	C6-XXM vs. C6-XYF	1.54674	0.95381	0

	B1-XXM vs. B1-XYM	1.51936	0.95791	0	C6-XXF vs. B1-XYM	2.49672	0.64626	0
	B1-XXM vs. B1-XYF	0.55727	0.99992	0	C6-XXF vs. B1-XYF	0.42009	0.99999	0
	B1-XXF vs. B1-XYM	3.89718	0.14074	1	C6-XXF vs. B1-XXM	0.97736	0.99671	0
	B1-XXF vs. B1-XYF	1.82055	0.89729	0	C6-XXF vs. B1-XXF	1.40046	0.97279	0
	B1-XXF vs. B1-XXM	2.37782	0.69859	0	C6-XXF vs. C6-XYM	1.44961	0.96719	0
	C6-XYM vs. B1-XYM	4.14637	0.09732	0	C6-XXF vs. C6-XYF	1.47925	0.96345	0
	C6-XYM vs. B1-XYF	1.90336	0.87443	0	C6-XXF vs. C6-XXM	0.06749	0.99999	0
**SP area (μm^2)**	B1-XYM<br>C6-XYM<br>B1-XYF<br>C6-XYF<br>B1-XXM<br>C6-XXM<br>B1-XXF<br>C6-XXF	6642.19 ± 788.04<br>5371.35 ± 367.49<br>7828.14 ± 534.78<br>6335.92 ± 794.09<br>8053.1 ± 691.61<br>6521.96 ± 692.34<br>5725.71 ± 400.03<br>6457.42 ± 680.38						
**SP area**	**2Way-ANOVA**	**F value**	**P value**	**Sig**	**Interactions (cont.)**	**q value**	**P value**	**Sig**
	Ab	$F_{(1,41)}$ = 4.11079	0.05051	0	C6-XYM vs. B1-XXM	2.57877	0.48723	0
	Genotype	$F_{(3,41)}$ = 2.28864	0.09605	0	C6-XYM vs. B1-XXF	0.60502	0.99985	0
	Interaction	$F_{(3,41)}$ = 1.4875	1.4875	0	C6-XYF vs. B1-XYM	0.48413	0.99997	0
	**Tukey Test**	**q value**			C6-XYF vs. B1-XYF	2.3588	0.70677	0
	Ab: B1 vs. C6	3.11735	0.03437	1	C6-XYF vs. B1-XXM	2.7144	0.54782	0
	Gen: XYF vs. XYM	2.75788	0.22689	0	C6-XYF vs. B1-XXF	0.96458	0.99697	0
	Gen: XXM vs. XYM	3.2377	0.12058	0	C6-XYF vs. C6-XYM	1.64688	0.93648	0
	Gen: XXM vs. XYF	0.45939	0.98795	0	C6-XXM vs. B1-XYM	0.19005	0.99999	0
	Gen: XXF vs. XYM	0.44526	0.989	0	C6-XXM vs. B1-XYF	2.06472	0.82246	0
	Gen: XXF vs. XYF	2.21417	0.41117	0	C6-XXM vs. B1-XXM	2.42032	0.6801	0
	Gen: XXF vs. XXM	2.67356	0.25104	0	C6-XXM vs. B1-XXF	1.25865	0.98504	0
	**Interactions**				C6-XXM vs. C6-XYM	1.96452	0.85585	0
	B1-XYF vs. B1-XYM	1.87467	0.88265	0	C6-XXM vs. C6-XYF	0.29408	0.99999	0
	B1-XXM vs. B1-XYM	2.23027	0.76012	0	C6-XXF vs. B1-XYM	0.29206	0.99999	0
	B1-XXM vs. B1-XYF	0.3556	0.99999	0	C6-XXF vs. B1-XYF	2.16673	0.78501	0
	B1-XXF vs. B1-XYM	1.44871	0.9673	0	C6-XXF vs. B1-XXM	2.52233	0.63478	0
	B1-XXF vs. B1-XYF	3.32337	0.2976	0	C6-XXF vs. B1-XXF	1.15664	0.99086	0
	B1-XXF vs. B1-XXM	3.67898	0.19043	0	C6-XXF vs. C6-XYM	1.85434	0.88828	0
	C6-XYM vs. B1-XYM	2.1698	0.78383	0	C6-XXF vs. C6-XYF	0.19207	0.99999	0
	C6-XYM vs. B1-XYF	4.19467	0.09037	0	C6-XXF vs. C6-XXM	0.10201	0.99999	0
**IZ area (μm^2)**	B1-XYM<br>C6-XYM<br>B1-XYF<br>C6-XYF<br>B1-XXM<br>C6-XXM<br>B1-XXF<br>C6-XXF	12223.76 ± 1001.93<br>10563.97 ± 981.41<br>9832.2 ± 447.84<br>9724.61 ± 551.61<br>10120.59 ± 626.01<br>10404.22 ± 418.44<br>10300.87 ± 327.19<br>11011.34 ± 446.19						
**IZ area**	**2Way-ANOVA**	**F value**	**P value**	**Sig**	**Interactions (cont.)**	**q value**	**P value**	**Sig**
	Ab	$F_{(1,41)}$ = 0.14959	0.70134	0	C6-XYM vs. B1-XXM	0.6653	0.99972	0
	Genotype	$F_{(3,41)}$ = 1.95545	0.13927	0	C6-XYM vs. B1-XXF	0.39479	0.99999	0
	Interaction	$F_{(3,41)}$ = 1.14063	0.34661	0	C6-XYF vs. B1-XYM	3.47181	0.24883	0
	**Tukey Test**	**q value**			C6-XYF vs. B1-XYF	0.14947	0.99999	0
	Ab: B1 vs. C6	0.5141	0.71846	0	C6-XYF vs. B1-XXM	0.5501	0.99992	0
	Gen: XYF vs. XYM	3.03107	0.16011	0	C6-XYF vs. B1-XXF	0.80054	0.99907	0
	Gen: XXM vs. XYM	2.03792	0.48337	0	C6-XYF vs. C6-XYM	1.25947	0.98499	0
	Gen: XXM vs. XYF	0.95087	0.9068	0	C6-XXM vs. B1-XYM	2.52771	0.63236	0
	Gen: XXF vs. XYM	1.23005	0.82032	0	C6-XXM vs. B1-XYF	0.79464	0.99912	0
	Gen: XXF vs. XYF	1.72434	0.61915	0	C6-XXM vs. B1-XXM	0.39401	0.99999	0
	Gen: XXF vs. XXM	0.77347	0.94674	0	C6-XXM vs. B1-XXF	0.14357	0.99999	0
	**Interactions**				C6-XXM vs. C6-XYM	0.23972	0.99999	0
	B1-XYF vs. B1-XYM	3.32234	0.29796	0	C6-XXM vs. C6-XYF	0.9441	0.99735	0
	B1-XXM vs. B1-XYM	2.92171	0.45573	0	C6-XXF vs. B1-XYM	1.68429	0.92903	0
	B1-XXM vs. B1-XYF	0.40063	0.99999	0	C6-XXF vs. B1-XYF	1.63805	0.93816	0
	B1-XXF vs. B1-XYM	2.67127	0.56736	0	C6-XXF vs. B1-XXM	1.23742	0.98644	0
	B1-XXF vs. B1-XYF	0.65107	0.99976	0	C6-XXF vs. B1-XXF	0.98698	0.99651	0
	B1-XXF vs. B1-XXM	0.25044	0.99999	0	C6-XXF vs. C6-XYM	0.67127	0.99651	0
	C6-XYM vs. B1-XYM	2.49051	0.64903	0	C6-XXF vs. C6-XYF	1.78752	0.90566	0
	C6-XYM vs. B1-XYF	1.09803	0.9933	0	C6-XXF vs. C6-XXM	0.84342	0.9987	0
**VZ area (μm^2)**	B1-XYM<br>C6-XYM<br>B1-XYF<br>C6-XYF<br>B1-XXM<br>C6-XXM<br>B1-XXF<br>C6-XXF	1778.73 ± 89.42<br>1534.88 ± 111.01<br>1507.21 ± 84.16<br>1243.95 ± 29.82<br>1385.14 ± 109.64<br>1714.03 ± 150.25<br>1416.24 ± 114.76<br>1562.82 ± 132.74						

VZ area	2Way-ANOVA	F value	P value	Sig	Interactions (cont.)	q value	P value	Sig
	Ab	$F_{(1,41)}$ = 0.01028	0.93879	0	C6-XYM vs. B1-XXM	1.43999	0.96835	0
	Genotype	$F_{(3,41)}$ = 2.34881	0.08985	0	C6-XYM vs. B1-XXF	1.14096	0.99157	0
	Interaction	$F_{(3,41)}$ = 3.52842	0.02502	1	C6-XYF vs. B1-XYM	2.76126	0.35761	0
	**Tukey Test**	**q value**			C6-XYF vs. B1-XYF	2.34382	0.71317	0
	Ab: B1 vs. C6	0.1094	0.93879	0	C6-XYF vs. B1-XXM	1.25703	0.98515	0
	Gen: XYF vs. XYM	3.43115	0.93879	0	C6-XYF vs. B1-XXF	1.53389	0.95576	0
	Gen: XXM vs. XYM	1.14282	0.85018	0	C6-XYF vs. C6-XYM	2.79774	0.51031	0
	Gen: XXM vs. XYF	2.19091	0.42042	0	C6-XXM vs. B1-XYM	0.57606	0.99989	0
	Gen: XXF vs. XYM	1.93262	0.52835	0	C6-XXM vs. B1-XYF	1.84138	0.89179	0
	Gen: XXF vs. XYF	1.43473	0.74213	0	C6-XXM vs. B1-XXM	2.92817	0.45294	0
	Gen: XXF vs. XXM	0.75617	0.94998	0	C6-XXM vs. B1-XXF	2.65131	0.57642	0
	**Interactions**				C6-XXM vs. C6-XYM	1.72279	0.92081	0
	B1-XYF vs. B1-XYM	2.41743	0.68137	0	C6-XXM vs. C6-XYF	4.1852	0.0917	0
	B1-XXM vs. B1-XYM	3.50422	0.23895	0	C6-XXF vs. B1-XYM	1.9223	0.86882	0
	B1-XXM vs. B1-XYF	1.08679	0.9937	0	C6-XXF vs. B1-XYF	0.49513	0.99996	0
	B1-XXF vs. B1-XYM	3.22737	0.33214	0	C6-XXF vs. B1-XXM	1.58192	0.94814	0
	B1-XXF vs. B1-XYF	0.80993	0.999	0	C6-XXF vs. B1-XXF	1.30507	0.98162	0
	B1-XXF vs. B1-XXM	0.27686	0.99999	0	C6-XXF vs. C6-XYM	0.26868	0.99999	0
	C6-XYM vs. B1-XYM	2.345	0.71266	0	C6-XXF vs. C6-XYF	2.83895	0.49197	0
	C6-XYM vs. B1-XYF	0.26613	0.99999	0	C6-XXF vs. C6-XXM	1.34625	0.97812	0

SUPPLEMENTAL TABLE 2: Statistical analysis for the behavioral assessments

SupTable2 Fig3B

**Fig. 3B: Open-field task ~ Time-in-center ~ 2Way repeated measures ANOVA**						
**Fig. 3B, top: Session 1 (S1): B1-XYM vs. C6-XYM**						
Tests of Within-Subjects Effects		***SS***	***DF***	***MS***	***F***	***Prob > F***
S1 time	Sphericity Assumed	176.19528	58	3.03785	1.64576	0.00219
	Greenhouse-Geisser	176.19528	9.11454	19.33123	1.64576	0.10724
S1 time * S1 Ab	Sphericity Assumed	102.43306	58	1.76609	0.95678	0.56852
	Greenhouse-Geisser	102.43306	9.11454	11.23843	0.95678	0.4793
Error (S1 time)	Sphericity Assumed	1605.90489	870	1.84587		
	Greenhouse-Geisser	1605.90489	136.7181	11.7461		
Tests of Between-Subjects Effects		***SS***	***DF***	***MS***	***F***	***Prob > F***
Intercept		821.21549	1	821.21549	127.42441	9.92352E-9
S1 Ab		0.28757	1	0.28757	0.04462	0.83555
Error		96.6709	15	6.44473		
S1-Ab descriptive statistics	***Mean***	***Std. Error***	***95.00% LCL***	***95.00% UCL***		
B1	0.93648	0.05364	0.8312	1.04177		
C6	0.90208	0.06411	0.77624	1.02792		
**Pairwise Comparison**	**Bonferroni Test**	***DF***	***|t| value***	***Prob > |t|***	***Sig Flag***	
B1 vs. C6		15	0.4116	0.68646	0	
**Fig. 3B, top: Session 2 (S2): B1-XYM vs. C6-XYM**						
Tests of Within-Subjects Effects		***SS***	***DF***	***MS***	***F***	***Prob > F***
S2 time	Sphericity Assumed	277.32081	58	4.78139	0.87506	0.73423
	Greenhouse-Geisser	277.32081	8.70301	31.86494	0.87506	0.54652
S2 time * S2 Ab	Sphericity Assumed	278.2319	58	4.7971	0.87793	0.72881
	Greenhouse-Geisser	278.2319	8.70301	31.96963	0.87793	0.54401
Error (S2 time)	Sphericity Assumed	4753.7443	870	5.46407		
	Greenhouse-Geisser	4753.7443	130.54511	36.41457		
Tests of Between-Subjects Effects		***SS***	***DF***	***MS***	***F***	***Prob > F***
Intercept		2515.89433	1	2515.89433	77.08479	2.68964E-7
S2 Ab		206.48999	1	206.48999	6.32667	0.02378
Error		489.57021	15	32.63801		
S2-Ab descriptive statistics	***Mean***	***Std. Error***	***95.00% LCL***	***95.00% UCL***		
B1	2.07	0.09413	1.88524	2.25476		
C6	1.14807	0.11251	0.92724	1.3689		
**Pairwise Comparison**	**Bonferroni Test**	***DF***	***|t| value***	***Prob > |t|***	***Sig Flag***	
B1 vs. C6		15	6.28488	1.46226E-5	1	
**Fig. 3B, middle: Session 1 (S1): B1-XYF vs. C6-XYF**						
Tests of Within-Subjects Effects		***SS***	***DF***	***MS***	***F***	***Prob > F***
S1 time	Sphericity Assumed	121.53159	58	2.09537	1.17886	0.17521
	Greenhouse-Geisser	121.53159	10.43246	11.64937	1.17886	0.30761
S1 time * S1 Ab	Sphericity Assumed	122.63185	58	2.11434	1.18953	0.16279
	Greenhouse-Geisser	122.63185	10.43246	11.75483	1.18953	0.30034
Error (S1 time)	Sphericity Assumed	1546.39017	870	1.77746		
	Greenhouse-Geisser	1546.39017	156.48696	9.88191		
Tests of Between-Subjects Effects		***SS***	***DF***	***MS***	***F***	***Prob > F***
Intercept		950.57219	1	950.57219	346.04002	8.9907E-12
S1 Ab		4.10536	1	4.10536	1.49449	0.24038
Error		41.20501	15	2.747		
S1-Ab descriptive statistics	***Mean***	***Std. Error***	***95.00% LCL***	***95.00% UCL***		
B1	1.05403	0.06191	0.93252	1.17555		
C6	0.92404	0.0518	0.82237	1.02571		

Pairwise Comparison	Bonferroni Test	***DF***	**|*t*| value**	**Prob > |*t*|**	***Sig Flag***	
B1 vs. C6		15	1.61048	0.12813	0	
**Fig. 3B, middle: Session 2 (S2): B1-XYF vs. C6-XYF**						
Tests of Within-Subjects Effects		***SS***	***DF***	***MS***	***F***	***Prob > F***
S2 time	Sphericity Assumed	302.30154	58	5.2121	1.33144	0.05361
	Greenhouse-Geisser	302.30154	8.38482	36.05345	1.33144	0.23128
S2 time * S2 Ab	Sphericity Assumed	397.76364	58	6.85799	1.75189	6.20878E-4
	Greenhouse-Geisser	397.76364	8.38482	47.43857	1.75189	0.08929
Error (S2 time)	Sphericity Assumed	3405.72828	870	3.91463		
	Greenhouse-Geisser	3405.72828	125.77224	27.07854		
Tests of Between-Subjects Effects		***SS***	***DF***	***MS***	***F***	***Prob > F***
Intercept		1663.23429	1	1663.23429	87.18813	1.22183E-7
S2 Ab		324.13246	1	324.13246	16.99129	9.04907E-4
Error		286.14575	15	19.07638		
S2-Ab descriptive statistics	***Mean***	***Std. Error***	***95.00% LCL***	***95.00% UCL***		
B1	1.8858	0.09441	1.7005	2.07111		
C6	0.73073	0.07899	0.57569	0.88576		
**Pairwise Comparison**	**Bonferroni Test**	***DF***	**|*t*| value**	**Prob > |*t*|**	***Sig Flag***	
B1 vs. C6		15	9.38401	1.14572E-7	1	
**Fig. 3B, bottom: Session 1 (S1): B1-XXM vs. C6-XXM**						
Tests of Within-Subjects Effects		***SS***	***DF***	***MS***	***F***	***Prob > F***
S1 time	Sphericity Assumed	171.89625	58	2.96373	1.43591	0.01981
	Greenhouse-Geisser	171.89625	12.42786	13.83153	1.43591	0.14795
S1 time * S1 Ab	Sphericity Assumed	98.05958	58	1.69068	0.81913	0.83076
	Greenhouse-Geisser	98.05958	12.42786	7.8903	0.81913	0.63453
Error (S1 time)	Sphericity Assumed	2154.81638	1044	2.064		
	Greenhouse-Geisser	2154.81638	223.70143	9.63256		
Tests of Between-Subjects Effects		***SS***	***DF***	***MS***	***F***	***Prob > F***
Intercept		1447.32289	1	1447.32289	238.41518	7.93255E-12
S1 Ab		12.7776	1	12.7776	2.10483	0.16404
Error		109.27078	18	6.0706		
S1-Ab descriptive statistics	***Mean***	***Std. Error***	***95.00% LCL***	***95.00% UCL***		
B1	1.00343	0.10144	0.79033	1.21654		
C6	1.21155	0.10144	0.99845	1.42466		
**Pairwise Comparison**	**Bonferroni Test**	***DF***	**|*t*| value**	**Prob > |*t*|**	***Sig Flag***	
B1 vs. C6		18	1.4508	0.16404	0	
**Fig. 3B, bottom: Session 2 (S2): B1-XXM vs. C6-XXM**						
Tests of Within-Subjects Effects		***SS***	***DF***	***MS***	***F***	***Prob > F***
S2 time	Sphericity Assumed	287.17387	58	4.95127	0.90639	0.67369
	Greenhouse-Geisser	287.17387	12.30955	23.32935	0.90639	0.5432
S2 time * S2 Ab	Sphericity Assumed	261.11766	58	4.50203	0.82415	0.82304
	Greenhouse-Geisser	261.11766	12.30955	21.2126	0.82415	0.62824
Error (S2 time)	Sphericity Assumed	5702.99619	1044	5.46264		
	Greenhouse-Geisser	5702.99619	221.57197	25.7388		
Tests of Between-Subjects Effects		***SS***	***DF***	***MS***	***F***	***Prob > F***
Intercept		4132.72985	1	4132.72985	159.55792	2.20018E-10
S2 Ab		46.30535	1	46.30535	1.78777	0.19785
Error		466.22028	18	25.90113		
S2-Ab descriptive statistics	***Mean***	***Std. Error***	***95.00% LCL***	***95.00% UCL***		
B1	1.67335	0.20952	1.23316	2.11354		
C6	2.06954	0.20952	1.62935	2.50974		
**Pairwise Comparison**	**Bonferroni Test**	***DF***	**|*t*| value**	**Prob > |*t*|**	***Sig Flag***	
B1 vs. C6		18	1.33708	0.19785	0	

SupTable2 Fig3C

**Fig. 3C: Open-field task ~ Time-in-center per subject ~ 3Way-ANOVA**							
**Test / Factors**	Statistic	P value			Statistic	P value	
**3Way-ANOVA**	**F value**	**P**	**Sig**	**Interactions (cont.)**	**q value**	**P**	**Sig**
Ab	$F_{(1,107)}$ = 7.43327	0.00761	1	B1-XYM-S1 vs. C6-XYF-S2	1.34595	0.99831	0
Genotype	$F_{(2,107)}$ = 4.21069	0.01767	1	B1-XYM-S1 vs. C6-XXM-S1	-1.79939	0.98067	0
Session	$F_{(1,107)}$ = 34.8721	5.31814E-8	1	B1-XYM-S1 vs. C6-XXM-S2	-7.41193	5.89153E-5	1
Ab * Genotype	$F_{(2,107)}$ = 9.01487	2.58315E-4	1	B1-XYM-S2 vs. B1-XYF-S1	6.03112	0.00262	1
Ab * Session	$F_{(1,107)}$ = 8.24853	0.00502	1	B1-XYM-S2 vs. B1-XYF-S2	1.09346	0.99976	0
Genotype * Session	$F_{(2,107)}$ = 1.87175	0.15942	0	B1-XYM-S2 vs. B1-XXM-S1	6.97696	2.05983E-4	1
Ab * Genotype * Session	$F_{(2,107)}$ = 3.91138	0.02328	1	B1-XYM-S2 vs. B1-XXM-S2	2.59469	0.79474	0
**Tukey Test**	**q value**			B1-XYM-S2 vs. C6-XYM-S1	6.93318	2.33057E-4	1
Ab: B1 vs. C6	4.0896	0.00474	1	B1-XYM-S2 vs. C6-XYM-S2	5.47289	0.01021	1
Gen: XYM vs. XYF	1.37138	0.59772	0	B1-XYM-S2 vs. C6-XYF-S1	7.49634	4.59803E-5	1
Gen: XYM vs. XXM	-2.80151	0.12242	0	B1-XYM-S2 vs. C6-XYF-S2	8.76088	1.03468E-6	1
Gen: XYF vs. XXM	-4.23771	0.00965	1	B1-XYM-S2 vs. C6-XXM-S1	5.61554	0.0073	1
Session: S1 vs. S2	-8.85789	5.06447E-8	1	B1-XYM-S2 vs. C6-XXM-S2	0.00301	0.9999	0
**Interactions**	**q value**			B1-XYF-S1 vs. B1-XYF-S2	-4.55231	0.07156	0
B1-XYM vs. B1-XYF	0.27976	0.99996	0	B1-XYF-S1 vs. B1-XXM-S1	0.30037	0.9999	0
B1-XYM vs. B1-XXM	1.52504	0.88883	0	B1-XYF-S1 vs. B1-XXM-S2	-3.67648	0.29473	0
B1-XYM vs. C6-XYM	4.01434	0.05989	0	B1-XYF-S1 vs. C6-XYM-S1	0.83165	0.99998	0
B1-XYM vs. C6-XYF	6.25244	3.64342E-4	1	B1-XYF-S1 vs. C6-XYM-S2	-0.51466	0.9999	0

B1-XYM vs. C6-XXM	-1.27023	0.94605	0	B1-XYM-S1 vs. C6-XYF-S1	0.77169	0.9999	0
B1-XXF vs. B1-XXM	1.10419	0.97016	0	B1-XYF-S1 vs. C6-XYF-S2	1.91924	0.96868	0
B1-XXF vs. C6-XYM	3.44311	0.15487	0	B1-XYF-S1 vs. C6-XXM-S1	-0.9351	0.99995	0
B1-XXF vs. B1-XYF	5.39424	0.00321	1	B1-XYF-S1 vs. C6-XXM-S2	-6.0284	0.00264	1
B1-XXF vs. C6-XYF	-1.43248	0.91258	0	B1-XYF-S2 vs. B1-XXM-S1	5.23804	0.01743	1
B1-XXM vs. C6-XXM	2.63039	0.43301	0	B1-XYF-S2 vs. B1-XXM-S2	1.26119	0.99907	0
B1-XXM vs. C6-XYM	4.7274	0.0146	1	B1-XYF-S2 vs. C6-XYM-S1	5.38396	0.01254	1
B1-XXM vs. C6-XYF	-2.79527	0.36335	0	B1-XYF-S2 vs. C6-XYM-S2	4.03764	0.00582	1
C6-XYM vs. C6-XXM	1.65966	0.84831	0	B1-XYF-S2 vs. C6-XYF-S1	5.70936	0.18432	0
C6-XYM vs. C6-XXM	-5.16706	0.00549	1	B1-XYF-S2 vs. C6-XYF-S2	6.85691	2.88638E-4	1
C6-XYF vs. C6-XXM	-7.52267	9.9748E-6	1	B1-XYF-S2 vs. C6-XXM-S1	4.00257	0.99976	0
B1-S1 vs. B1-S2	-9.30972	1.15E-15	1	B1-XYF-S2 vs. C6-XXM-S2	-1.09073	0.09776	0
B1-S1 vs. C6-S1	-0.15446	0.99953	0	B1-XXM-S1 vs. B1-XXM-S2	-4.38227	0.09776	0
B1-S1 vs. C6-S2	-3.37169	0.08702	0	B1-XXM-S1 vs. C6-XYM-S1	0.60169	0.9999	0
B1-S2 vs. C6-S1	9.15526	1.15E-15	1	B1-XXM-S1 vs. C6-XYM-S2	-0.8586	0.99998	0
B1-S2 vs. C6-S2	5.93803	3.45259E-4	1	B1-XXM-S1 vs. C6-XYF-S1	0.51937	0.9999	0
C6-S1 vs. C6-S2	-3.21723	0.11115	0	B1-XXM-S1 vs. C6-XYF-S2	1.78392	0.98192	0
XYM-S1 vs. XYM-S2	-5.79066	0.00121	1	B1-XXM-S1 vs. C6-XXM-S1	-1.36142	0.99813	0
XYM-S1 vs. XYF-S1	-0.58561	0.9984	0	B1-XXM-S1 vs. C6-XXM-S2	-6.97396	2.0774E-4	1
XYM-S1 vs. XYF-S2	-3.26563	0.20057	0	B1-XXM-S2 vs. C6-XYM-S1	4.57853	0.0681	0
XYM-S1 vs. XXM-S1	-1.6548	0.84989	0	B1-XXM-S2 vs. C6-XYM-S2	3.11825	0.5503	0
XYM-S1 vs. XXM-S2	-8.37152	7.54987E-7	1	B1-XXM-S2 vs. C6-XYF-S1	4.90164	0.03588	1
XYM-S2 vs. XYF-S1	5.20505	0.00502	1	B1-XXM-S2 vs. C6-XYF-S2	6.16619	0.00185	1
XYM-S2 vs. XYF-S2	2.52503	0.47992	0	B1-XXM-S2 vs. C6-XXM-S1	3.02085	0.59878	0
XYM-S2 vs. XXM-S1	4.40959	0.02819	0	B1-XXM-S2 vs. C6-XXM-S2	-2.59169	0.79594	0
XYM-S2 vs. XXM-S2	-2.30714	0.58006	0	C6-XYM-S1 vs. C6-XYM-S2	-1.34632	0.99831	0
XYF-S1 vs. XYF-S2	-2.68002	0.41151	0	C6-XYM-S1 vs. C6-XYF-S1	-0.13037	0.9999	0
XYF-S1 vs. XXM-S1	-1.0415	0.97687	0	C6-XYM-S1 vs. C6-XYF-S2	1.01719	0.99988	0
XYF-S1 vs. XXM-S2	-7.75823	4.92314E-6	1	C6-XYM-S1 vs. C6-XXM-S1	-1.83716	0.97736	0
XYF-S2 vs. XXM-S1	1.7652	0.81186	0	C6-XYM-S1 vs. C6-XXM-S2	-6.93045	2.34853E-4	1
XYF-S2 vs. XXM-S2	-4.95153	0.00895	1	C6-XYM-S2 vs. C6-XYF-S1	1.32992	0.99848	0
XXM-S1 vs. XXM-S2	-7.0674	3.79097E-5	1	C6-XYM-S2 vs. C6-XYF-S2	2.47748	0.83904	0
B1-XYM-S1 vs. B1-XYM-S2	-7.41493	5.83991E-5	1	C6-XYM-S2 vs. C6-XXM-S1	-0.37687	0.9999	0
B1-XYM-S1 vs. B1-XYF-S1	-0.69782	0.9999	0	C6-XYM-S2 vs. C6-XXM-S2	-5.47017	0.01028	1
B1-XYM-S1 vs. B1-XYF-S2	-5.63549	0.00696	1	C6-XYF-S1 vs. C6-XYF-S2	1.26454	0.99904	0
B1-XYM-S1 vs. B1-XXM-S1	-0.43797	0.9999	0	C6-XYF-S1 vs. C6-XXM-S1	-1.88079	0.97301	0
B1-XYM-S1 vs. B1-XXM-S2	-4.82024	0.04238	1	C6-XYF-S1 vs. C6-XXM-S2	-7.49333	4.63891E-5	1
B1-XYM-S1 vs. C6-XYM-S1	0.20424	0.9999	0	C6-XYF-S2 vs. C6-XXM-S1	-3.14534	0.53682	0
B1-XYM-S1 vs. C6-XYM-S2	-1.25605	0.9991	0	C6-XYF-S2 vs. C6-XXM-S2	-8.75787	1.04371E-6	1
B1-XYM-S1 vs. C6-XYF-S1	0.08141	0.9999	0	C6-XXM-S1 vs. C6-XXM-S2	-5.61254	0.00735	1

SupTable2 Fig3D

**Fig. 3D: Open-field task ~ S2 – S1 difference ~ 2Way-ANOVA**								
**Variable**	**Test / Factors**	Statistic	P value			Statistic	P value	
**S2 - S1 Diff**	**2Way-ANOVA**	**F value**	**P**	**Sig**	**Interactions (cont.)**	**q value**	**P**	**Sig**
	Ab	$F_{(1,53)}$ = 10.58954	0.00209	1	C6-XYM vs. B1-XYM	4.26543	0.04437	1
	Genotype	$F_{(2,53)}$ = 2.40298	0.10125	0	C6-XYM vs. B1-XYF	2.4217	0.53061	0
	Interaction	$F_{(2,53)}$ = 5.02146	0.01047	1	C6-XYM vs. B1-XXM	1.90093	0.75911	0
	**Tukey Test**	**q value**			C6-XYF vs. B1-XYM	6.5562	3.77876E-4	1
	Ab: B1 vs. C6	4.65749	0.00186	1	C6-XYF vs. B1-XYF	4.59659	0.02428	1
	Gen: XYF vs. XYM	3.4744	0.04575	1	C6-XYF vs. B1-XXM	3.97978	0.0723	0
	Gen: XXM vs. XYM	0.02756	0.99979	0	C6-XYF vs. C6-XYM	1.96988	0.73097	0
	Gen: XXM vs. XYF	3.58495	0.03803	1	C6-XXM vs. B1-XYM	1.36147	0.92737	0
	**Interactions**	**q value**			C6-XXM vs. B1-XYF	0.11756	0.9999	0
	B1-XYF vs. B1-XYM	1.35307	0.92913	0	C6-XXM vs. B1-XXM	0.92931	0.98571	0
	B1-XXM vs. B1-XYM	2.29078	0.58987	0	C6-XXM vs. C6-XYM	2.74426	0.39102	0
	B1-XXM vs. B1-XYF	0.72578	0.99541	0	C6-XXM vs. C6-XYF	5.19473	0.00749	1

SupTable2 Fig4A_top

**Fig. 4A, TOP: Open-field task: Movement ~ 3Way-ANOVA**							
**Test / Factors**	Statistic	P value			Statistic	P value	
3**Way-ANOVA**	**F value**	**P**	**Sig**	**Interactions (cont.)**	**q value**	**P**	**Sig**
Ab	$F_{(1,107)}$ = 0.63832	0.42629	0	B1-XYM-S1 vs. C6-XYF-S2	16.4827	1.111E-15	1
Genotype	$F_{(2,107)}$ = 11.04104	4.83269E-5	1	B1-XYM-S1 vs. C6-XXM-S1	1.21593	0.99934	0
Session	$F_{(1,107)}$ = 191.72	1.111E-15	1	B1-XYM-S1 vs. C6-XXM-S2	8.0261	9.42392E-6	1
Ab * Genotype	$F_{(2,107)}$ = 4.36555	0.01532	1	B1-XYM-S2 vs. B1-XYF-S1	-6.05993	0.00244	1
Ab * Session	$F_{(1,107)}$ = 4.9395	0.0286	1	B1-XYM-S2 vs. B1-XYF-S2	1.00487	0.99989	0
Genotype * Session	$F_{(2,107)}$ = 5.32223	0.00643	1	B1-XYM-S2 vs. B1-XXM-S1	-6.20676	0.00167	1
Ab * Genotype * Session	$F_{(2,107)}$ = 0.89161	0.41336	0	B1-XYM-S2 vs. B1-XXM-S2	-0.73568	0.99999	0
**Tukey Test**	**q value**			B1-XYM-S2 vs. C6-XYM-S1	-9.67398	7.25098E-8	1
Ab: B1 vs. C6	1.19843	0.39887	0	B1-XYM-S2 vs. C6-XYM-S2	0.85487	0.99998	0
Gen: XYM vs. XYF	6.5314	3.54836E-5	1	B1-XYM-S2 vs. C6-XYF-S1	-6.23598	0.00155	1
Gen: XYM vs. XXM	1.16194	0.69059	0	B1-XYM-S2 vs. C6-XYF-S2	6.91011	2.48677E-4	1
Gen: XYF vs. XXM	-5.6782	3.44561E-4	1	B1-XYM-S2 vs. C6-XXM-S1	-8.35666	3.45283E-6	1
Session: S1 vs. S2	20.76945	4.00452E-8	1	B1-XYM-S2 vs. C6-XXM-S2	-1.54649	0.99431	0
**Interactions**	**q value**			B1-XYF-S1 vs. B1-XYF-S2	6.51342	7.41258E-4	1
B1-XYM vs. B1-XYF	2.56816	0.46054	0	B1-XYF-S1 vs. B1-XXM-S1	0.42739	0.99999	0

B1-XYM vs. B1-XXM	1.8598	0.77604	0	B1-XYF-S1 vs. B1-XXM-S2	5.39231	0.0123	1
B1-XYM vs. C6-XYM	-0.09343	0.99999	0	B1-XYF-S1 vs. C6-XYM-S1	-3.33199	0.44555	0
B1-XYM vs. C6-XYF	7.24552	2.2639E-5	1	B1-XYF-S1 vs. C6-XYM-S2	6.37513	0.00107	1
B1-XYM vs. C6-XXM	-0.23374	0.99998	0	B1-XYM-S1 vs. C6-XYF-S1	0.40087	0.99999	0
B1-XXF vs. B1-XXM	-0.88042	0.98911	0	B1-XYF-S1 vs. C6-XYF-S2	12.33075	6.94605E-9	1
B1-XXF vs. C6-XYM	-2.45386	0.51233	0	B1-XYF-S1 vs. C6-XXM-S1	-1.52362	0.99499	0
B1-XXF vs. B1-XYF	4.00705	0.06069	0	B1-XYF-S1 vs. C6-XXM-S2	4.65652	0.05864	0
B1-XXF vs. C6-XYM	-2.78027	0.36946	0	B1-XYF-S2 vs. B1-XXM-S1	-6.63741	5.2944E-4	1
B1-XXM vs. C6-XYM	-1.78117	0.80601	0	B1-XYF-S2 vs. B1-XXM-S2	-1.67249	0.98914	0
B1-XXM vs. C6-XYF	5.38572	0.00328	1	B1-XYF-S2 vs. C6-XYM-S1	-9.84541	5.04689E-8	1
B1-XXM vs. C6-XYM	-2.09354	0.67751	0	B1-XYF-S2 vs. C6-XYM-S2	-0.14673	0.99999	0
C6-XYM vs. C6-XYF	6.66863	1.17163E-4	1	B1-XYF-S2 vs. C6-XYF-S1	-6.66393	4.92377E-4	1
C6-XYM vs. C6-XXM	-0.11868	0.99999	0	B1-XYF-S2 vs. C6-XYF-S2	-0.13829	0.99999	0
C6-XYF vs. C6-XXM	-7.47926	1.13484E-5	1	B1-XYF-S2 vs. C6-XXM-S1	-8.58841	1.71802E-6	1
B1-S1 vs. B1-S2	12.3289	1.111E-15	1	B1-XYF-S2 vs. C6-XXM-S2	-2.40828	0.86251	0
B1-S1 vs. C6-S1	-1.5099	0.70996	0	B1-XXM-S1 vs. B1-XXM-S2	5.47108	0.01025	1
B1-S1 vs. C6-S2	15.53363	3.15023E-8	1	B1-XXM-S1 vs. C6-XYM-S1	-4.04144	0.17345	0
B1-S2 vs. C6-S1	-13.8388	1.111E-15	1	B1-XXM-S1 vs. C6-XYM-S2	6.48741	7.95024E-4	1
B1-S2 vs. C6-S2	3.20473	0.11332	0	B1-XXM-S1 vs. C6-XYF-S1	-0.02923	0.99999	0
C6-S1 vs. C6-S2	17.04353	1.74367E-7	1	B1-XXM-S1 vs. C6-XYF-S2	13.11686	2.20205E-9	1
XYM-S1 vs. XYM-S2	13.58764	0.02163	1	B1-XXM-S1 vs. C6-XXM-S1	-2.1499	0.93131	0
XYM-S1 vs. XYF-S1	4.69658	5.63367E-9	1	B1-XXM-S1 vs. C6-XXM-S2	4.66027	0.05821	0
XYM-S1 vs. XYF-S2	18.12785	0.64459	0	B1-XXM-S2 vs. C6-XYM-S1	-9.00636	4.54777E-7	1
XYM-S1 vs. XXM-S1	3.80995	1.111E-15	1	B1-XXM-S2 vs. C6-XYM-S2	1.52249	0.99502	0
XYM-S1 vs. XXM-S2	12.06321	0.96963	0	B1-XXM-S2 vs. C6-XYF-S1	-5.5003	0.00958	1
XYM-S2 vs. XYF-S1	-8.89106	3.03414E-5	1	B1-XXM-S2 vs. C6-XYF-S2	7.64579	2.95421E-5	1
XYM-S2 vs. XYF-S2	4.54021	1.111E-15	1	B1-XXM-S2 vs. C6-XXM-S1	-7.62098	3.18024E-5	1
XYM-S2 vs. XXM-S1	-10.41999	5.75275E-5	1	B1-XXM-S2 vs. C6-XXM-S2	-0.81081	0.99999	0
XYM-S2 vs. XXM-S2	-2.16672	2.88962E-7	1	C6-XYM-S1 vs. C6-XYM-S2	9.70712	6.72866E-8	1
XYF-S1 vs. XYF-S2	13.43127	0.78158	0	C6-XYM-S1 vs. C6-XYF-S1	4.01492	0.18081	0
XYF-S1 vs. XXM-S1	-1.10865	6.9626E-8	1	C6-XYM-S1 vs. C6-XYF-S2	15.9448	1.111E-15	1
XYF-S1 vs. XXM-S2	7.14462	0.42951	0	C6-XYM-S1 vs. C6-XXM-S1	2.09044	0.94297	0
XYF-S2 vs. XXM-S1	-15.17481	8.32395E-7	1	C6-XYM-S1 vs. C6-XXM-S2	8.27057	4.4842E-6	1
XYF-S2 vs. XXM-S2	-6.92154	0.99991	0	C6-XYM-S2 vs. C6-XYF-S1	-6.51393	7.40241E-4	1
XXM-S1 vs. XXM-S2	8.68416	9.96999E-8	1	C6-XYM-S2 vs. C6-XYF-S2	5.41595	0.01165	1
B1-XYM-S1 vs. B1-XYM-S2	9.57259	9.23275E-8	1	C6-XYM-S2 vs. C6-XXM-S1	-8.43841	2.6958E-6	1
B1-XYM-S1 vs. B1-XYF-S1	2.62705	0.78158	0	C6-XYM-S2 vs. C6-XXM-S2	-2.25828	0.9061	0
B1-XYM-S1 vs. B1-XYF-S2	9.69185	6.9626E-8	1	C6-XYF-S1 vs. C6-XYF-S2	13.14609	2.03316E-9	1
B1-XYM-S1 vs. B1-XXM-S1	3.36583	0.42951	0	C6-XYF-S1 vs. C6-XXM-S1	-2.12068	0.93723	0
B1-XYM-S1 vs. B1-XXM-S2	8.83691	8.32395E-7	1	C6-XYF-S1 vs. C6-XXM-S2	4.6895	0.05499	0
B1-XYM-S1 vs. C6-XYM-S1	-0.987	0.99991	0	C6-XYF-S2 vs. C6-XXM-S1	-15.26677	1.111E-15	1
B1-XYM-S1 vs. C6-XYM-S2	9.54185	9.96999E-8	1	C6-XYF-S2 vs. C6-XXM-S2	-8.45659	2.55179E-6	1
B1-XYM-S1 vs. C6-XYF-S1	3.33661	0.44335	0	C6-XXM-S1 vs. C6-XXM-S2	6.81017	3.28807E-4	1

SupTable2 Fig4A_bottom

**Fig. 4A, BOTTOM: Open-field task: Distance ~ 3Way-ANOVA**							
**Test / Factors**	Statistic	P value			Statistic	P value	
3**Way-ANOVA**	**F value**	**P**	**Sig**	**Interactions (cont.)**	**q value**	**P**	**Sig**
Ab	$F_{(1,107)}$ = 0.67441	0.41347	0	B1-XYM-S1 vs. C6-XYF-S2	11.69214	1.04317E-8	1
Genotype	$F_{(2,107)}$ = 3.34683	0.03918	1	B1-XYM-S1 vs. C6-XXM-S1	-0.65889	0.99999	0
Session	$F_{(1,107)}$ = 143.661	1.111E-15	1	B1-XYM-S1 vs. C6-XXM-S2	6.95008	2.1213E-4	1
Ab * Genotype	$F_{(2,107)}$ = 2.68676	0.07302	0	B1-XYM-S2 vs. B1-XYF-S1	-6.80617	3.18476E-4	1
Ab * Session	$F_{(1,107)}$ = 2.35927	0.1277	0	B1-XYM-S2 vs. B1-XYF-S2	-1.25212	0.99913	0
Genotype * Session	$F_{(2,107)}$ = 1.79977	0.17065	0	B1-XYM-S2 vs. B1-XXM-S1	-7.31556	7.38204E-5	1
Ab * Genotype * Session	$F_{(2,107)}$ = 1.79977	0.17065	0	B1-XYM-S2 vs. B1-XXM-S2	-2.02675	0.95397	0
**Tukey Test**	**q value**			B1-XYM-S2 vs. C6-XYM-S1	-8.46428	2.22088E-6	1
Ab: B1 vs. C6	1.2291	0.38687	0	B1-XYM-S2 vs. C6-XYM-S2	0.58786	0.99999	0
Gen: XYM vs. XYF	2.20751	0.26737	0	B1-XYM-S2 vs. C6-XYF-S1	-6.48931	7.62949E-4	1
Gen: XYM vs. XXM	-1.51574	0.53374	0	B1-XYM-S2 vs. C6-XYF-S2	2.482	0.83761	0
Gen: XYF vs. XXM	-3.86094	0.02028	1	B1-XYM-S2 vs. C6-XXM-S1	-9.47692	1.11032E-7	1
Session: S1 vs. S2	17.9388	1.2201E-8	1	B1-XYM-S2 vs. C6-XXM-S2	-1.86795	0.97445	0
**Interactions**	**q value**			B1-XYF-S1 vs. B1-XYF-S2	5.12058	0.02226	1
B1-XYM vs. B1-XYF	-0.03964	0.99999	0	B1-XYF-S1 vs. B1-XXM-S1	0.16741	0.99999	0
B1-XYM vs. B1-XXM	-0.37072	0.99983	0	B1-XYF-S1 vs. B1-XXM-S2	4.96692	0.03096	1
B1-XYM vs. C6-XYM	0.08896	0.99999	0	B1-XYF-S1 vs. C6-XYM-S1	-1.52871	0.99487	0
B1-XYM vs. C6-XYF	3.67896	0.10645	0	B1-XYF-S1 vs. C6-XYM-S2	6.81696	3.08979E-4	1
B1-XYM vs. C6-XXM	-1.78675	0.80399	0	B1-XYM-S1 vs. C6-XYF-S1	1.21019	0.99937	0
B1-XXF vs. B1-XXM	-0.29678	0.99994	0	B1-XYF-S1 vs. C6-XYF-S2	9.28699	1.83431E-7	1
B1-XXF vs. C6-XYM	0.11856	1	0	B1-XYF-S1 vs. C6-XXM-S1	-1.794	0.98119	0
B1-XXF vs. B1-XYF	3.35321	0.17653	0	B1-XYF-S1 vs. C6-XXM-S2	5.11103	0.02273	1
B1-XXF vs. C6-XYM	-1.58181	0.87264	0	B1-XYF-S2 vs. B1-XXM-S1	-5.38664	0.01225	1
B1-XXM vs. C6-XYM	0.42538	0.99966	0	B1-XYF-S2 vs. B1-XXM-S2	-0.58712	0.99999	0
B1-XXM vs. C6-XYF	4.06616	0.05417	0	B1-XYF-S2 vs. C6-XYM-S1	-6.64928	4.92661E-4	1
B1-XXM vs. C6-XYM	-1.41603	0.91649	0	B1-XYF-S2 vs. C6-XYM-S2	1.69638	0.98788	0
C6-XYM vs. C6-XYF	3.21995	0.21334	0	B1-XYF-S2 vs. C6-XYF-S1	-4.54484	0.07203	0
C6-XYM vs. C6-XXM	-1.71041	0.83131	0	B1-XYF-S2 vs. C6-XYF-S2	3.53196	0.3538	0

C6-XYF vs. C6-XXM	-5.54515	0.00218	1	B1-XYF-S2 vs. C6-XXM-S1	-7.34805	6.7102E-5	1
B1-S1 vs. B1-S2	10.92388	1.111E-15	1	B1-XYF-S2 vs. C6-XXM-S2	-0.44302	0.99999	0
B1-S1 vs. C6-S1	-0.75644	0.95035	0	B1-XXM-S1 vs. B1-XXM-S2	5.28881	0.01531	1
B1-S1 vs. C6-S2	13.55375	1.111E-15	1	B1-XXM-S1 vs. C6-XYM-S1	-1.82552	0.97851	0
B1-S2 vs. C6-S1	-11.81555	1.111E-15	1	B1-XXM-S1 vs. C6-XYM-S2	7.22662	9.56672E-5	1
B1-S2 vs. C6-S2	2.49464	0.29679	0	B1-XXM-S1 vs. C6-XYF-S1	1.15154	0.99961	0
C6-S1 vs. C6-S2	14.49183	1.111E-15	1	B1-XXM-S1 vs. C6-XYF-S2	10.12285	3.11625E-8	1
XYM-S1 vs. XYM-S2	12.05927	1.111E-15	1	B1-XXM-S1 vs. C6-XXM-S1	-2.16137	0.92905	0
XYM-S1 vs. XYF-S1	2.89415	0.32402	0	B1-XXM-S1 vs. C6-XXM-S2	5.44761	0.01064	1
XYM-S1 vs. XYF-S2	12.49939	1.111E-15	1	B1-XXM-S2 vs. C6-XYM-S1	-6.62504	5.26687E-4	1
XYM-S1 vs. XXM-S1	0.90907	0.98742	0	B1-XXM-S2 vs. C6-XYM-S2	2.42711	0.85651	0
XYM-S1 vs. XXM-S2	9.57665	2.22045E-8	1	B1-XXM-S2 vs. C6-XYF-S1	-4.37244	0.09889	0
XYM-S2 vs. XYF-S1	-9.3775	3.5854E-8	1	B1-XXM-S2 vs. C6-XYF-S2	4.59887	0.06499	0
XYM-S2 vs. XYF-S2	0.22775	0.99998	0	B1-XXM-S2 vs. C6-XXM-S1	-7.45017	4.96381E-5	1
XYM-S2 vs. XXM-S1	-11.72024	1.111E-15	1	B1-XXM-S2 vs. C6-XXM-S2	0.1588	0.99999	0
XYM-S2 vs. XXM-S2	-3.05265	0.26657	0	C6-XYM-S1 vs. C6-XYM-S2	8.34567	3.28842E-6	1
XYF-S1 vs. XYF-S2	9.78058	1.4452E-8	1	C6-XYM-S1 vs. C6-XYF-S1	2.92831	0.64433	0
XYF-S1 vs. XXM-S1	-2.10951	0.67036	0	C6-XYM-S1 vs. C6-XYF-S2	11.00511	1.57654E-8	1
XYF-S1 vs. XXM-S2	6.72593	9.56914E-5	1	C6-XYM-S1 vs. C6-XXM-S1	-0.13589	0.99999	0
XYF-S2 vs. XXM-S1	-12.18613	1.111E-15	1	C6-XYM-S1 vs. C6-XXM-S2	6.76915	3.53236E-4	1
XYF-S2 vs. XXM-S2	-3.35069	0.17718	0	C6-XYM-S2 vs. C6-XYF-S1	-6.45141	8.4526E-4	1
XXM-S1 vs. XXM-S2	9.12011	7.18634E-8	1	C6-XYM-S2 vs. C6-XYF-S2	1.62539	0.99143	0
B1-XYM-S1 vs. B1-XYM-S2	8.81803	7.37798E-7	1	C6-XYM-S2 vs. C6-XXM-S1	-9.18803	2.42692E-7	1
B1-XYM-S1 vs. B1-XYF-S1	1.19607	0.99943	0	C6-XYM-S2 vs. C6-XXM-S2	-2.283	0.89979	0
B1-XYM-S1 vs. B1-XYF-S2	6.75011	3.72502E-4	1	C6-XYF-S1 vs. C6-XYF-S2	9.40919	1.31985E-7	1
B1-XYM-S1 vs. B1-XXM-S1	1.50248	0.99557	0	C6-XYF-S1 vs. C6-XXM-S1	-3.40902	0.40896	0
B1-XYM-S1 vs. B1-XXM-S2	6.79128	3.32033E-4	1	C6-XYF-S1 vs. C6-XXM-S2	4.5383	0.07293	0
B1-XYM-S1 vs. C6-XYM-S1	-0.46205	0.99999	0	C6-XYF-S2 vs. C6-XXM-S1	-12.38033	5.67349E-9	1
B1-XYM-S1 vs. C6-XYM-S2	8.5901	1.49895E-6	1	C6-XYF-S2 vs. C6-XXM-S2	-4.43301	0.08864	0
B1-XYM-S1 vs. C6-XYF-S1	2.72083	0.74154	0	C6-XXM-S1 vs. C6-XXM-S2	7.60897	3.09267E-5	1

SupTable2 Fig4B

**Fig. 4B: Self-grooming ~ 2Way-ANOVA**								
**Variable**	**Test / Factors**	Statistic	P value			Statistic	P value	
**Time (s)**	**2Way-ANOVA**	**F value**	**P**	**Sig**	**Interactions (cont.)**	**q value**	**P**	**Sig**
	Ab	$F_{(1,55)}$ = 1.24515	0.26982	0	C6-XYM vs. B1-XYM	0.41983	0.99967	0
	Genotype	$F_{(2,55)}$ = 0.42262	0.65764	0	C6-XYM vs. B1-XYF	1.78033	0.80541	0
	Interaction	$F_{(2,55)}$ = 5.02146	0.27298	0	C6-XYM vs. B1-XXM	1.10264	0.96973	0
	**Tukey Test**	**q value**			C6-XYF vs. B1-XYM	0.20989	0.99999	0
	Ab: B1 vs. C6	1.40134	0.00186	0	C6-XYF vs. B1-XYF	1.75486	0.81463	0
	Gen: XYF vs. XYM	0.90702	0.79807	0	C6-XYF vs. B1-XXM	0.99577	0.9806	0
	Gen: XXM vs. XYM	0.90044	0.80066	0	C6-XYF vs. C6-XYM	0.24606	0.99998	0
	Gen: XXM vs. XYF	0.80066	0.99991	0	C6-XXM vs. B1-XYM	0.195	0.99999	0
	**Interactions**	**q value**			C6-XXM vs. B1-XYF	1.33426	0.93307	0
	B1-XYF vs. B1-XYM	1.51121	0.89142	0	C6-XXM vs. B1-XXM	0.55743	0.9987	0
	B1-XXM vs. B1-XYM	0.75242	0.99458	0	C6-XXM vs. C6-XYM	0.59678	0.99819	0
	B1-XXM vs. B1-XYF	0.8284	0.99155	0	C6-XXM vs. C6-XYF	0.41356	0.99969	0

SupTable2 Fig4C

**Fig. 4C: Marbles buried ~ 2Way-ANOVA**								
**Variable**	**Test / Factors**	Statistic	P value			Statistic	P value	
**Number (#)**	**2Way-ANOVA**	**F value**	**P**	**Sig**	**Interactions (cont.)**	**q value**	**P**	**Sig**
	Ab	$F_{(1,64)}$ = 0.10411	0.7481	0	C6-XYM vs. B1-XYM	0.32093	0.99991	0
	Genotype	$F_{(2,64)}$ = 13.79503	1.2157E-5	1	C6-XYM vs. B1-XYF	2.25657	0.60464	0
	Interaction	$F_{(2,64)}$ = 0.13556	0.8735	0	C6-XYM vs. B1-XXM	0.23484	0.55542	0
	**Tukey Test**	**q value**			C6-XYF vs. B1-XYM	3.18975	0.22889	0
	Ab: B1 vs. C6	4.65749	0.00186	0	C6-XYF vs. B1-XYF	0.47646	0.9994	0
	Gen: XYF vs. XYM	3.91738	0.02017	0	C6-XYF vs. B1-XXM	0.01054	2.82965E-4	1
	Gen: XXM vs. XYM	3.51313	0.04134	0	C6-XYF vs. C6-XYM	3.04935	0.27378	0
	Gen: XXM vs. XYF	7.78448	2.48557E-6	1	C6-XXM vs. B1-XYM	2.36181	0.5566	0
	**Interactions**	**q value**			C6-XXM vs. B1-XYF	4.2426	0.04353	1
	B1-XYF vs. B1-XYM	2.21523	0.62342	0	C6-XXM vs. B1-XXM	0.7192	0.99565	0
	B1-XXM vs. B1-XYM	3.17017	0.23484	0	C6-XXM vs. C6-XYM	1.70643	0.83191	0
	B1-XXM vs. B1-XYF	4.97006	0.01054	1	C6-XXM vs. C6-XYF	5.59834	0.0027	1

SupTable2 Fig5B

**Fig. 5B: Three-chamber task ~ Sociability ~ 3Way-ANOVA**							
**Test / Factors**	Statistic	P value			Statistic	P value	
3**Way-ANOVA**	**F value**	**P**	**Sig**	**Interactions (cont.)**	**q value**	**P**	**Sig**
Ab	$F_{(1,125)}$ = 3.73586	0.05574	0	B1-XYM-ms vs. C6-XXM-ob	5.14516	0.02023	1
Genotype	$F_{(2,125)}$ = 8.06283	5.30751E-4	1	B1-XYM-ms vs. C6-XYF-ms	-7.82574	1.29375E-5	1
Stimulus	$F_{(1,125)}$ = 212.94611	1.111E-15	1	B1-XYM-ms vs. C6-XYF-ob	5.09658	0.02254	1
Ab * Genotype	$F_{(2,125)}$ = 0.46553	0.62899	0	B1-XYM-ob vs. B1-XXM-ms	-9.27992	1.36744E-7	1
Ab * Stimulus	$F_{(1,125)}$ = 4.0954	0.04534	0	B1-XYM-ob vs. B1-XXM-ob	0.04278	0.99999	0
Genotype * Stimulus	$F_{(2,125)}$ = 11.67322	2.44334E-5	1	B1-XYM-ob vs. B1-XYF-ms	-8.84873	4.9337E-7	1
Ab * Genotype * Stimulus	$F_{(2,125)}$ = 1.27723	0.28277	0	B1-XYM-ob vs. B1-XYF-ob	0.45555	0.99999	0
**Tukey Test**	**q value**			B1-XYM-ob vs. C6-XYM-ms	-4.74337	0.04763	1
Ab: B1 vs. C6	-2.87371	0.04448	0	B1-XYM-ob vs. C6-XYM-ob	-0.31284	0.99999	0

Gen: XYM vs. XYF	-4.91738	0.00206	1	B1-XYM-ob vs. C6-XXM-ms	-13.64419	4.38311E-9	1
Gen: XYM vs. XXM	-5.57535	4.07943E-4	1	B1-XYM-ob vs. C6-XXM-ob	0.61977	0.99999	0
Gen: XYF vs. XXM	0.27937	0.97872	0	B1-XYM-ob vs. C6-XYF-ms	-12.43238	1.09342E-8	1
Stimulus: ms vs. ob	21.69615	3.60612E-8	1	B1-XYM-ob vs. C6-XYF-ob	0.48993	0.99999	0
**Interactions**	**q value**			B1-XXM-ms vs. B1-XXM-ob	10.21251	2.83235E-8	1
B1-XYM vs. B1-XXM	-3.22246	0.21141	0	B1-XXM-ms vs. B1-XYF-ms	-1.11998	0.9997	0
B1-XYM vs. B1-XYF	-3.15461	0.23204	0	B1-XXM-ms vs. B1-XYF-ob	8.78518	6.03089E-7	1
B1-XYM vs. C6-XYM	-0.79501	0.99322	0	B1-XXM-ms vs. C6-XYM-ms	3.25051	0.48381	0
B1-XYM vs. C6-XXM	-6.00972	6.15872E-4	0	B1-XXM-ms vs. C6-XYM-ob	7.96717	8.28277E-6	1
B1-XYM vs. C6-XYF	-5.1872	0.00491	0	B1-XXM-ms vs. C6-XXM-ms	-5.08351	0.0232	1
B1-XXM vs. B1-XYF	-0.47608	0.99941	0	B1-XXM-ms vs. C6-XXM-ob	10.44139	2.45979E-8	1
B1-XXM vs. C6-XYM	2.03591	0.70285	0	B1-XXM-ms vs. C6-XYF-ms	-3.60128	0.32284	0
B1-XXM vs. C6-XXM	-3.14944	0.23367	0	B1-XXM-ms vs. C6-XYF-ob	10.51065	2.37669E-8	1
B1-XXM vs. C6-XYF	-2.20069	0.62887	0	B1-XXM-ob vs. B1-XYF-ms	-9.45846	8.66719E-8	1
B1-XYF vs. C6-XYM	2.17545	0.64043	0	B1-XXM-ob vs. B1-XYF-ob	0.4467	0.99999	0
B1-XYF vs. C6-XXM	-2.14174	0.65575	0	B1-XXM-ob vs. C6-XYM-ms	-5.08797	0.02297	1
B1-XYF vs. C6-XYF	-1.33795	0.9335	0	B1-XXM-ob vs. C6-XYM-ob	-0.37131	0.99999	0
C6-XYM vs. C6-XXM	-4.58673	0.01888	0	B1-XXM-ob vs. C6-XXM-ms	-14.89537	1.111E-15	1
C6-XYM vs. C6-XYF	-3.81834	0.08312	0	B1-XXM-ob vs. C6-XXM-ob	0.62953	0.99999	0
C6-XXM vs. C6-XYF	0.97761	0.9826	1	B1-XXM-ob vs. C6-XYF-ms	-13.62289	4.49439E-9	1
B1-ms vs. B1-ob	13.09598	1.111E-15	1	B1-XXM-ob vs. C6-XYF-ob	0.48904	0.99999	0
B1-ms vs. C6-ms	-4.15958	0.02038	1	B1-XYF-ms vs. B1-XYF-ob	8.57812	1.16935E-6	1
B1-ms vs. C6-ob	13.30948	1.111E-15	1	B1-XYF-ms vs. C6-XYM-ms	3.78496	0.25172	0
B1-ob vs. C6-ms	-17.37352	2.79252E-7	1	B1-XYF-ms vs. C6-XYM-ob	7.86971	1.12675E-5	1
B1-ob vs. C6-ob	0.09554	0.99989	0	B1-XYF-ms vs. C6-XXM-ms	-3.11482	0.55136	0
C6-ms vs. C6-ob	17.62929	8.96192E-7	1	B1-XYF-ms vs. C6-XXM-ob	9.72692	4.99942E-8	1
XYM-ms vs. XYM-ob	5.91313	7.95671E-4	1	B1-XYF-ms vs. C6-XYF-ms	-1.85286	0.97621	0
XYM-ms vs. XXM-ms	-8.57816	2.77876E-7	1	B1-XYF-ms vs. C6-XYF-ob	9.74127	4.87929E-8	1
XYM-ms vs. XXM-ob	7.22885	1.90234E-5	1	B1-XYF-ob vs. C6-XYM-ms	-4.79316	0.04303	1
XYM-ms vs. XYF-ms	-7.81392	3.12934E-6	1	B1-XYF-ob vs. C6-XYM-ob	-0.70841	0.99999	0
XYM-ms vs. XYF-ob	6.9185	4.79721E-5	1	B1-XYF-ob vs. C6-XXM-ms	-12.75579	9.13996E-9	1
XYM-ob vs. XXM-ms	-15.11358	4.22541E-9	1	B1-XYF-ob vs. C6-XXM-ob	0.08594	0.99999	0
XYM-ob vs. XXM-ob	0.69342	0.99643	0	B1-XYF-ob vs. C6-XYF-ms	-11.63342	1.55376E-8	1
XYM-ob vs. XYF-ms	-13.87272	7.44763E-9	1	B1-XYF-ob vs. C6-XYF-ob	-0.03929	0.99999	0
XYM-ob vs. XYF-ob	0.85969	0.99028	0	C6-XYM-ms vs. C6-XYM-ob	4.08475	0.1593	0
XXM-ms vs. XXM-ob	17.91579	1.111E-15	1	C6-XYM-ms vs. C6-XXM-ms	-7.36875	5.31201E-5	1
XXM-ms vs. XYF-ms	0.15409	0.99999	0	C6-XYM-ms vs. C6-XXM-ob	5.47299	0.00949	1
XXM-ms vs. XYF-ob	16.52768	8.3946E-10	1	C6-XYM-ms vs. C6-XYF-ms	-6.16838	0.00164	1
XXM-ob vs. XYF-ms	-16.13259	1.44723E-9	1	C6-XYM-ms vs. C6-XYF-ob	5.42575	0.01062	1
XXM-ob vs. XYF-ob	0.241	0.99998	0	C6-XYM-ob vs. C6-XXM-ms	-11.95961	1.36243E-8	1
XYF-ms vs. XYF-ob	15.11419	4.22389E-9	1	C6-XYM-ob vs. C6-XXM-ob	0.88213	0.99997	0
B1-XYM-ms vs. B1-XYM-ob	4.33273	0.10429	0	C6-XYM-ob vs. C6-XYF-ms	-10.82571	2.08564E-8	1
B1-XYM-ms vs. B1-XXM-ms	-4.60003	0.06331	0	C6-XYM-ob vs. C6-XYF-ob	0.76843	0.99999	0
B1-XYM-ms vs. B1-XXM-ob	4.72267	0.04966	1	C6-XXM-ms vs. C6-XXM-ob	14.96017	1.111E-15	1
B1-XYM-ms vs. B1-XYF-ms	-4.91684	0.03325	1	C6-XXM-ms vs. C6-XYF-ms	1.53067	0.99489	0
B1-XYM-ms vs. B1-XYF-ob	4.38744	0.09449	0	C6-XXM-ms vs. C6-XYF-ob	15.10833	1.111E-15	1
B1-XYM-ms vs. C6-XYM-ms	-0.81148	0.99999	0	C6-XXM-ob vs. C6-XYF-ms	-13.72577	3.82671E-9	1
B1-XYM-ms vs. C6-XYM-ob	3.61906	0.31551	0	C6-XXM-ob vs. C6-XYF-ob	-0.14811	0.99999	0
B1-XYM-ms vs. C6-XXM-ms	-9.1188	2.15987E-7	1	C6-XYF-ms vs. C6-XYF-ob	13.85764	3.82671E-9	1

SupTable2 Fig5C

**Fig. 5C: Three-chamber task ~ Discrimination Ratio ~ 2Way-ANOVA**								
**Variable**	**Test / Factors**	Statistic	P value			Statistic	P value	
**Time (s)**	**2Way-ANOVA**	**F value**	**P**	**Sig**	**Interactions (cont.)**	**q value**	**P**	**Sig**
	Ab	$F_{(1,62)}$ = 0.90927	0.34433	0	C6-XYM vs. B1-XYM	0.10235	0.99999	0
	Genotype	$F_{(2,62)}$ = 7.28274	0.00153	0	C6-XYM vs. B1-XYF	2.82012	0.35841	0
	Interaction	$F_{(2,62)}$ = 0.41749	0.6607	0	C6-XYM vs. B1-XXM	2.41548	0.53234	0
	**Tukey Test**	**q value**			C6-XYF vs. B1-XYM	4.30097	0.03951	1
	Ab: B1 vs. C6	2.37032	0.0992	0	C6-XYF vs. B1-XYF	0.75107	0.99467	0
	Gen: XXM vs. XYM	4.61133	0.00526	1	C6-XYF vs. B1-XXM	1.92482	0.74961	0
	Gen: XYF vs. XYM	5.17338	0.00159	1	C6-XYF vs. C6-XYM	3.9665	0.07118	0
	Gen: XYF vs. XXM	0.90213	0.7999	0	C6-XXM vs. B1-XYM	4.26381	0.04228	0
	**Interactions**	**q value**			C6-XXM vs. B1-XYF	0.77519	0.99382	0
	B1-XYF vs. B1-XYM	2.9565	0.3068	0	C6-XXM vs. B1-XXM	1.92666	0.74887	0
	B1-XXM vs. B1-XYM	2.57877	0.45931	0	C6-XXM vs. C6-XYM	3.94473	0.07384	0
	B1-XXM vs. B1-XYF	0.84091	0.991	0	C6-XXM vs. C6-XYF	0.04139	0.99999	0
